# Preadipocytes in human granulation tissue: role in wound healing and response to macrophage polarization

**DOI:** 10.1186/s41232-023-00302-5

**Published:** 2023-10-31

**Authors:** Tina Rauchenwald, Florian Handle, Catherine E. Connolly, Antonia Degen, Christof Seifarth, Martin Hermann, Christoph H. Tripp, Doris Wilflingseder, Susanne Lobenwein, Dragana Savic, Leo Pölzl, Evi M. Morandi, Dolores Wolfram, Ira-Ida Skvortsova, Patrizia Stoitzner, Johannes Haybaeck, Marko Konschake, Gerhard Pierer, Christian Ploner

**Affiliations:** 1 Department of Plastic, Reconstructive and Aesthetic Surgery, Medical University of Innsbruck, Innsbruck, Austria; 2 Institute of Pathology, Neuropathology and Molecular Pathology, Medical University Innsbruck, Innsbruck, Austria; 3 Institute of Clinical and Functional Anatomy, Medical University of Innsbruck, Innsbruck, Austria; 4 Department of Anesthesiology and Critical Care Medicine, Medical University of Innsbruck, Innsbruck, Austria; 5 Department of Dermatology, Venereology and Allergology, Medical University of Innsbruck, Innsbruck, Austria; 6 Institute of Hygiene and Medical Microbiology, Medical University of Innsbruck, Innsbruck, Austria; 7 Department of Therapeutic Radiology and Oncology, Medical University of Innsbruck, EXTRO-Lab, Tyrolean Cancer Research Institute, Innsbruck, Austria; 8 Department of Cardiac Surgery, Medical University of Innsbruck, Innsbruck, Austria; 9 Diagnostic and Research Center for Molecular Biomedicine, Institute of Pathology, Medical University of Graz, Graz, Austria

**Keywords:** Preadipocytes, Adipose-derived stem cells, Macrophage polarization, Granulation tissue, Wound healing, Inflammation, Tissue fibrosis, Myofibroblasts

## Abstract

**Background:**

Chronic non-healing wounds pose a global health challenge. Under optimized conditions, skin wounds heal by the formation of scar tissue. However, deregulated cell activation leads to persistent inflammation and the formation of granulation tissue, a type of premature scar tissue without epithelialization. Regenerative cells from the wound periphery contribute to the healing process, but little is known about their cellular fate in an inflammatory, macrophage-dominated wound microenvironment.

**Methods:**

We examined CD45^−^/CD31^−^/CD34^+^ preadipocytes and CD68^+^ macrophages in human granulation tissue from pressure ulcers (*n*=6) using immunofluorescence, immunohistochemistry, and flow cytometry. In vitro, we studied macrophage-preadipocyte interactions using primary human adipose-derived stem cells (ASCs) exposed to conditioned medium harvested from IFNG/LPS (M1)- or IL4/IL13 (M2)-activated macrophages. Macrophages were derived from THP1 cells or CD14^+^ monocytes. In addition to confocal microscopy and flow cytometry, ASCs were analyzed for metabolic (OXPHOS, glycolysis), morphological (cytoskeleton), and mitochondrial (ATP production, membrane potential) changes. Angiogenic properties of ASCs were determined by HUVEC-based angiogenesis assay. Protein and mRNA levels were assessed by immunoblotting and quantitative RT-PCR.

**Results:**

CD45^−^/CD31^−^/CD34^+^ preadipocytes were observed with a prevalence of up to 1.5% of total viable cells in human granulation tissue. Immunofluorescence staining suggested a spatial proximity of these cells to CD68^+^ macrophages in vivo. In vitro, ASCs exposed to M1, but not to M2 macrophage secretome showed a pro-fibrotic response characterized by stress fiber formation, elevated alpha smooth muscle actin (SMA), and increased expression of integrins ITGA5 and ITGAV. Macrophage-secreted IL1B and TGFB1 mediated this response via the PI3K/AKT and p38-MAPK pathways. In addition, ASCs exposed to M1-inflammatory stress demonstrated reduced migration, switched to a glycolysis-dominated metabolism with reduced ATP production, and increased levels of inflammatory cytokines such as IL1B, IL8, and MCP1. Notably, M1 but not M2 macrophages enhanced the angiogenic potential of ASCs.

**Conclusion:**

Preadipocyte fate in wound tissue is influenced by macrophage polarization. Pro-inflammatory M1 macrophages induce a pro-fibrotic response in ASCs through IL1B and TGFB1 signaling, while anti-inflammatory M2 macrophages have limited effects. These findings shed light on cellular interactions in chronic wounds and provide important information for the potential therapeutic use of ASCs in human wound healing.

**Supplementary Information:**

The online version contains supplementary material available at 10.1186/s41232-023-00302-5.

## Background

Human wound healing is a highly efficient process that requires the spatial and temporal synergy of a multifaceted system [1]. The interaction and coordination of multiple cell types during the sequential wound-healing steps of inflammation, proliferation, and remodeling are critical for skin repair [2–4].

Macrophages play a central role in all stages of tissue repair by exerting regulatory activity on tissue integrity [5]. Macrophages are classified into pro-inflammatory M1 macrophages and anti-inflammatory M2 macrophages according to their phenotype upon activation by microenvironmental stimuli, known as the M1/M2 polarization process [6, 7]. The acute phase of wound healing is dominated by M1 macrophages, which are activated by damage- and pathogen-associated molecular patterns (DAMPs and PAMPs) to clear injured tissue from cellular debris and invading pathogens. As levels of DAMPs and PAMPs decrease, M2 macrophages replace M1 macrophages to promote tissue remodeling for re-epithelialization and scar formation [8, 9]. In addition to their phagocytic function, macrophages are an important source of cytokines that affect adjacent cells in the wound tissue [10]. Inflammatory interleukins (IL) and members of the tumor growth factor (TGF) family are known cytokines secreted by pro-inflammatory M1 macrophages [10] that induce activation of intracellular kinase cascades including protein kinase B (AKT/PKB), mitogen-activated protein kinases (p38-MAPK), and extracellular signal-regulated protein kinase (ERK1/2) [11, 12]. In wound tissue, this inflammation results in the recruitment of dermal fibroblasts and their subsequent transition to a myofibrotic phenotype characterized by increased stress fiber formation, elevated levels of alpha-smooth muscle actin (SMA), and altered cell secretome [13–15]. Whether macrophages exert similar effects on other resident wound tissue cell types is unknown.

In skin repair, failure to transition from the M1 phenotype to the M2 phenotype results in impaired wound healing and failure to progress through the stages of tissue repair [3, 4, 16]. Pathological wound healing is prolonged and can lead to chronic non-healing wounds. At the molecular level, the chronic wound environment is dominated by persistent inflammatory signaling [17]. While dermal substructure formation and re-epithelialization are impeded, a premature form of scar tissue, named granulation tissue, is established to protect the underlying structures. The cellularity of granulation tissue is complex and the literature on this topic is scarce. A recent study investigating the cellularity of diabetic foot ulcers (DFUs) using single-cell RNA sequencing identified up to 21 different cell types in DFU wounds [18].

Interestingly, the authors noted the presence of fibroblast subtypes that were associated with healing progression and may be responsible for altered rates of tissue repair. Whether these cells are derived from dermal fibroblast progenitors [19–21] that become activated upon injury, or from tissue-infiltrating regenerative cells from neighboring adipose tissue remains elusive. In support of the second possibility, lineage-tracing experiments in mice have identified wound-infiltrating CD34^+^/CD29^+^/Sca1^+^ adipocyte precursor cells in injured tissue [22]. Like tissue-resident quiescent skin fibroblasts and invading dedifferentiated adipocytes, these cells contributed to the myofibroblast population present in healing wounds [22]. However, the molecular mechanisms regulating the fate and function of CD34^+^ cells remain unknown and their presence in human wounds has not been described so far.

In the present study, we report the presence of CD45^−^/CD31^−^/CD34^+^ preadipocytes in human granulation tissue samples and show the spatial proximity of these cells to CD68^+^ macrophages. We investigate the effect of an inflammatory wound bed environment dominated by IFNG/LPS-activated macrophages on the physiology of adipose-derived stem cells (ASCs) as model for CD45^-^/CD31^-^/CD34^+^ preadipocytes and show that macrophage polarization is the determining factor for a pro-fibrotic fate of ASCs. 

## Material and methods

### Granulation tissue harvest and cell isolation

Granulation tissue was obtained from chronic wounds of six different donors (mean age 43.3 ± 22.8 years; 33.3% female, 66.7% male, *n* = 6). Wound etiology was pressure ulcer in all cases. All patients underwent wound treatment with a vacuum-assisted closure dressing device (3M^TM^ V.A.C.® Dressing, KCI, Minnesota, USA) prior to defect coverage. Tissue samples were obtained at the time of defect coverage surgery. Patient and wound characteristics are shown in Supplement Table 1. Written informed consent was obtained from all participating donors, and ethical approval was granted by the Ethics Committee of the Medical University of Innsbruck (EK0244/2018, EK4368/2016).

Immunohistochemistry and immunofluorescence were performed on representative tissue sections. Immunofluorescence microscopy was carried out with a spinning disk confocal Operetta CLS System (PerkinElmer, Waltham, MA, USA). To prepare a single-cell suspension, the remaining tissue sample was minced into small pieces and digested in collagenase type I (Roche, Germany) 0.3% in RPMI-1640 (PAN Biotech, Germany) supplemented with 100 ng/ml DNAse (Merck, Austria) for 3 h at 37°C. To accelerate digestion, the mixture was thoroughly vortexed every 15 min. The digested granulation tissue was then filtered through a 100-µm sterile strainer (Corning Incorporated - Life Sciences, Germany), pelleted, and resuspended in erythrocyte lysis buffer (BioLegend, Germany) for 5 min (min) at RT°C. Cells were again filtered through a 40-µm sterile strainer (Corning Incorporated - Life Sciences, Germany), counted, and stored at −80°C in FCS supplemented with 10% DMSO before FACS analysis.

### Isolation of human adipose-derived stem cells (ASCs)

Isolation of human ASCs was performed according to our previously published protocol [23, 24]. Abdominal subcutaneous adipose tissue was obtained from patients (mean age 37.2 ± 13.1 years; 60% female, 40% male; *n* = 25) undergoing elective body-contouring surgery after major weight loss. All patients signed written informed consent and the study was approved by the Ethics Committee of the Medical University of Innsbruck (EK0244/2018, EK4368/2016).

For ASC isolation, superficial subcutaneous adipose tissue was washed with phosphate-buffered saline (PBS, PAN-Biotech GmbH, Germany), minced into small pieces, and digested by incubation with collagenase type I (0.15% in PBS) for 1 h at 37°C with gentle agitation. The samples were filtered through a mesh and centrifuged at 300 *g* for 5 min. The supernatant was discarded, and the pelleted stromal vascular fraction (SVF) was subjected to two cycles of PBS washing followed by centrifugation at 300 *g* for 5 min. Erythrocyte lysis buffer (RBC Lysis Buffer (10X), BioLegend, Germany) was applied for 20 min, and samples were filtered through a 100-µm cell strainer (Corning Incorporated - Life Sciences, Germany) and centrifuged at 300 *g* for 5 min. The pelleted SVF was resuspended in DMEM-F12 medium (PAN-Biotech GmbH, Germany) and then filtered through a 40-µm cell strainer (Corning Incorporated - Life Sciences, Germany). Cells were counted using a CASY cell counter (CASY Model TTC, Schärfe-System GmbH, Germany) and plated at a density of 10,000 cells/cm^2^ in PM4 medium [25] consisting of DMEM-F12 medium (PAN-Biotech GmbH, Germany) supplemented with 1 ng/ml rhFGF2 (Immunotools, Germany), 10 ng/ml EGF (Immunotools, Germany), 50 ng/ml Insulin (Roche, Switzerland), 2.5% FCS, 1% penicillin/streptomycin (GE Healthcare, Austria), and 1% gentamycin (Sigma-Aldrich, Austria). After 1 h of incubation at 37°C, non-adherent cells were discarded and PM4 medium was added for cell culturing in a humidified 5% CO_2_ incubator at 37°C.

### Generation of macrophage-conditioned media (MQ-CM)

The generation of macrophage-conditioned media (MQ-CM) was based on our previously published method [23]. THP1 cells (ATCC® TIB-202™), human monocytic cells derived from a patient with acute monocytic leukemia, were cultured in RPMI-1640 medium (PAN-Biotech GmbH, Germany) supplemented with 10% FCS, 1% penicillin/streptomycin (GE Healthcare, Austria), and 1% gentamycin (Sigma-Aldrich, Austria). For THP1 maturation, the medium was changed to DMEM-F12 medium (PAN-Biotech GmbH, Germany) supplemented with 2.5% FCS, 1% penicillin/streptomycin (GE Healthcare, Austria), and 1% gentamycin (Sigma-Aldrich, Austria). According to our standardized maturation procedure, maturation was induced by the addition of 25 ng/ml PMA (Sigma-Aldrich, Austria) dissolved in sterile filtered EtOH for 24 h, followed by a 24-h recovery period before differential macrophage activation. Differential macrophage activation was induced for 24 h in DMEM-F12 + 2.5% FCS by the addition of 10 ng/ml recombinant human interferon-gamma (IFNG) (PeproTech, Austria) and 10 ng/ml *E. coli* lipopolysaccharide (LPS) (Sigma-Aldrich, Austria) for differentiation of pro-inflammatory M(IFNG/LPS) (= M1) or by addition of 10 ng/ml interleukin 4 (IL4) (PeproTech, Austria) and 10 ng/ml interleukin 13 (IL13) (PeproTech, Austria) for differentiation of anti-inflammatory M(IL4/IL13) (= M2). After cytokine stimulation, cells were washed and cultured in DMEM-F12 + 2.5% FCS for another 24 h to obtain conditioned media (CM). Untreated THP1 cells (= Mo) without PMA stimulation and THP1 cells stimulated with PMA alone for 24 h (= MΦ) were cultured in DMEM-F12 + 2.5% FCS without additional stimulatory signals for the same period. CM of untreated THP1 cells, PMA-stimulated THP1 cells, and differentially activated macrophages (M(IFNG/LPS) and M(IL4/IL13)) were harvested, centrifuged at 300 *g* for 5 min, and filtered through a 0.2-µm sieve (Sarstedt, Germany) to remove residual cellular components. CM of untreated THP1 cells was labeled as Mo-CM, CM of PMA-stimulated THP1 cells was labeled as MΦ-CM, CM of (M(IFNG/LPS)) was labeled as M1-CM, and CM from M(IL4/IL13)) was labeled as M2-CM. CM was stored at −80°C.

### Culturing and treatment of human ASCs

ASCs were plated and cultured in PM4 medium for 24 h followed by 24 h starvation in DMEM-F12 only. Cells were treated with MQ-CM from differentially activated macrophages for 72 h, including conditioned medium from monocytic THP1 cells (Mo-CM), quiescent (inactive) macrophages (MΦ-CM), pro-inflammatory, IFNG/LPS-stimulated macrophages (M1-CM), and anti-inflammatory, IL4/IL13-stimulated macrophages (M2-CM). ASCs treated with DMEM-F12 supplemented with 2.5% FCS served as control (ctr). Additional different treatments of cultured ASCs were administered according to the same preparation protocol and included 10 ng/ml recombinant human transforming growth factor beta 1 (TGFB1) (PeproTech, Austria) and 10 ng/ml recombinant human interleukin 1 beta (IL1B) (PeproTech, Austria). The study of M1-CM-induced effects was complemented by different co-treatments. M1-CM was co-treated with either IL1 receptor antagonist IL1RA (100 ng/ml) (PeproTech, Austria), TGFB1 receptor kinase inhibitor SB431542 (1 µM) (Bio-Techne, Ireland), PI3K/AKT inhibitor LY294002 (25 µM) (Calbiochem®, California, USA), ERK1/2 inhibitor U0126 (10 µM) (Merck Millipore, Germany), or p38 MAPK inhibitor Losmapimod (GW856553X; 25 µM) (Selleck Chemicals GmbH, Germany). After 72 h of incubation, cells were harvested for further analysis.

### Monocyte isolation and macrophage differentiation

The generation of macrophage-conditioned media from CD14^+^ monocyte-derived macrophages was described previously [23]. Briefly, monocytes were isolated from the blood of healthy donors using CD14 BD IMAG Beads (Becton-Dickinson, BD, Austria) according to the manufacturer’s instructions. Written informed consent was obtained from all participating blood donors by the Central Institute for Blood Transfusion & Immunology Department, Innsbruck, Austria. Approval for the use of anonymized residual samples for scientific purposes was granted by the Ethics Committee of the Medical University of Innsbruck (EK1166/2018). Monocytes were resuspended in RPMI-1640 medium (PAN Biotech, Germany) containing 5% AB serum and 2-mM l-glutamine and cultured with GMCSF (50 ng/ml) or MCSF (50 ng/ml) at 37°C/5% CO_2_ for 7 days. GMCSF-stimulated macrophages were further challenged with 10 ng/ml recombinant human IFNG (PeproTech, Austria) and 10 ng/ml E.coli LPS (Sigma-Aldrich, Missouri, USA) for 24 h to obtain pro-inflammatory GMCSF (IFNG/LPS) macrophages. MCSF-stimulated macrophages were challenged with 10ng/ml recombinant human IL4 (PeproTech, Austria) and 10 ng/ml recombinant human IL13 (PeproTech, Austria) to obtain anti-inflammatory MCSF (IL4/IL13) macrophages. After stimulation, cells were washed three times with PBS to remove activator cytokines and cultured in RPMI-1640/2.5% FCS for another 24 h to generate conditioned media (CM). Finally, CM was harvested, centrifuged at 300 *g* for 10 min, and filtered (pore size < 0.2 µm) to remove any residual cellular components.

### Flow cytometry

Single-cell suspensions of human granulation tissue samples were resuspended in PBS and stained with Fixable Viability Dye eFluor® 780 (Thermo Fisher Scientific, Austria) according to the manufacturer’s instructions. Cells were washed thoroughly in PBS and resuspended in Brilliant Staining Buffer (BD Biosciences, Germany) diluted 1:2 with PBS for labeling. Endothelial progenitor cells (EPCs) and adipose-derived stem cells (ASCs) were stained with monoclonal antibodies against the following surface markers: CD45-FITC (clone HI30, BioLegend, Germany), CD31-PE/Cy7 (clone WM-59, BioLegend, Germany), CD34-PE (clone 561, BioLegend, Germany), and CD90-BV510 (clone eBio5E10, Thermo Fisher Scientific, Austria). Stained cells were acquired using CytoFLEX S flow cytometer (Beckman Coulter Life Science, Germany), and flow cytometry data were analyzed using Flowjo™ analysis software (Version 10.8.1, BD Bioscience). Cell death of MQ-CM-treated ASCs was assessed by AnnexinV-FITC and 7AAD staining according to the manufacturer’s instructions.

### Immunohistochemistry

Human granulation tissue samples were fixed in formaldehyde (4%) and embedded in paraffin-wax. Sections of 2.5-µm thickness were mounted on TOMO® microscope slides (Matsunami Glass, USA). Immunohistochemistry was performed using Ventana Benchmark Ultra Immunostainer (Roche, Germany).

Primary antibodies used were anti-CD31 (rabbit monoclonal, ab28364, Abcam, UK) ready-to-use without antigen retrieval and anti-CD34 (mouse monoclonal, QBEnd10, Dako, Denmark) ready-to-use with standard antigen retrieval using Ultra Cell Conditioning Solution (Ultra CC1-Ventana, Roche, Germany).

### Immunofluorescence

For immunofluorescence, sections were deparaffinized and incubated with MaxBlock Autofluorescence Reducing Reagent Kit (MB-M, MaxVision Biosciences, USA) according to the manufacturer’s instructions. Slides were pretreated with TRIS/EDTA buffer at pH 9 for 18 min at 97°C and blocked with 5% donkey serum (ab7475, Abcam, UK) to prevent unspecific binding. Antibody incubation was performed overnight at 4°C using primary antibodies against CD34 (anti-CD34, mouse monoclonal, 134M-18, Cell Marque™, Sigma-Aldrich®, Austria), CD31 (anti-CD31, rabbit monoclonal, ab28364, Abcam, UK), and CD68 (anti-CD68, rabbit monoclonal, CS76347, Cell Signaling Technology, The Netherlands).

Primary antibodies were visualized with donkey serum-conjugated secondary antibodies: anti-mouse Alexa Fluor Plus 555 (A32773, Invitrogen, California, USA) and anti-rabbit Alexa Fluor Plus 488 (A32790, Invitrogen, California, USA). After extensive washing, the slides were counterstained with HOECHST-33342 and coverslipped with VECTASHIELD mounting solution (Vector Laboratories, H-1000-10, California, USA). Images were captured using the Operetta CLS™ from Perkin Elmer and analyzed using Harmony™ software.

### Public datasets

The publicly available single-cell RNA sequencing dataset from Theocharidis et al. [18] was downloaded from the NCBI-GEO repository (accession number: GSE165816). Bioinformatic analysis of the downloaded count matrix files was performed in R (Version 4.2.0) using the following packages: Seurat (Version 4.2.0), MAST (Version 1.22.0) and Nebulosa (Version 1.6.0). Poor-quality cells were removed based on the detected gene number (<400 and >4000) and mitochondrial gene content (>10 %). Normalization, integration, and dimension reduction were performed using the SCTransform/RPCA/UMAP pipeline. Cell types were annotated after KNN clustering using the markers published in the original study. Gene expression was visualized with violin plots using weighted kernel density estimation for UMAP plots.

### Microscopy of phalloidin and vinculin staining

To visualize cytoskeletal stress fibers and focal adhesion formation, cells were fixed in 3% paraformaldehyde (PFA), permeabilized with 0.5% Triton-X 100 (VWR, Germany) in PBS, blocked with 1% bovine serum albumin (BSA) (Sigma-Aldrich, Austria) in PBS for 1 h, and stained with vinculin (1:200, Vinculin eFLuor® 570, Clone 7F9, Thermo Fisher Scientific, Austria), phalloidin (1:1500, Phalloidin Cruz Fluor™ 488 Conjugate, Szabo Scandic, Austria), and Hoechst (1:1000, Hoechst 33342, Thermo Fischer Scientific, Austria). Microscopic images were acquired using a spinning disk confocal system (UltraVIEW VoX; Perkin Elmer, Waltham, MA) attached to a Zeiss AxioObserver Z1 microscope (Zeiss, Oberkochen, Germany).

### Cell metabolic analysis

The Agilent Seahorse XFp Extracellular Flux Analyzer (Agilent Technologies, California, USA) was used to measure two major energy-producing cellular pathways: mitochondrial respiration and glycolysis. The Cell Energy Phenotype Test was performed on live ASCs under baseline conditions and under stressed conditions. Equal numbers of cells (3000/well) were plated in Seahorse XFp Cell Culture Miniplates Analyzer (Agilent Technologies, California, USA) and incubated in a growth medium at 37°C and 5% CO_2_ overnight. Cells were treated the next day. XFp cartridges were hydrated in a non-CO_2_ incubator at 37°C overnight the day before the assay. On the day of analysis, the cell medium was changed to XF base medium (Agilent Technologies, California, USA) supplemented with 10 mM of glucose, 2 mM of l-glutamine, and 1 mM of sodium pyruvate. The pH was adjusted to 7.4 and incubation at 37°C, non-CO_2_ for 1 h prior to the assay was performed. Cells were then exposed to a “stressor mix,” a simultaneous injection of oligomycin (an inhibitor of mitochondrial ATP synthase) and FCCP (a mitochondrial uncoupling agent) with final well concentrations of 2 µM and 1 µM, respectively. The assay consisted of three basal and five exercise measurements. In all cases, oxygen consumption rate (OCR), which evaluates mitochondrial respiration, and extracellular acidification rate (ECAR), which reveals pH changes associated with glycolytic activity, were determined. Background OCR and ECAR values were obtained from wells without cells (medium only) using XFp software and automatically subtracted.

The obtained results were normalized to the cell number. After the assay, cells were stained with DAPI solution, images of whole wells were captured by a Lionheart FX automated microscope (Agilent BioTek), and cell numbers were evaluated using the open-access ImageJ/Fiji software. Data analysis and reporting were performed using Seahorse Wave Desktop software and the Seahorse XF Cell Energy Phenotype Test Report Generator.

### Assessment of mitochondrial membrane potential and ATP measurement

ASCs pretreated with differentially activated MQ-CM for 72 h as described above were analyzed for mitochondrial membrane potential using the lipophilic cation tetramethylrhodamine methyl ester (TMRM) (VWR, Germany). Cells were incubated for 20 min at 37°C in DMEM-F12 medium supplemented with 2.5% FCS and 100 nM TMRM. For adenosine triphosphate (ATP) measurement, pretreated ASCs were incubated for 20 min at 37°C with prewarmed HBSS medium supplemented with ATP-Red (Thermo Fisher Scientific, Austria). Cell analysis was performed on a BD FACSCalibur^TM^ flow cytometer (BD Biosciences, Germany), and data were analyzed using Flowjo^TM^ analysis software (version 10.8.1, BD Biosciences, Germany).

### Angiogenesis assay

The angiogenesis assay was performed using human umbilical vein endothelial cells (HUVECs) cultured primarily on gelatin-coated plates in EGMTM Endothelial Cell Growth Medium BulletKitTM (EBMTM Basal Medium (CC-3121) and EGMTM Endothelial Cell Growth Medium SingleQuotsTM Supplements (CC-4133), Lonza, Switzerland) as previously described [26]. Approval was obtained from the Ethics Committee of the Medical University of Innsbruck (UN4435). CM from pretreated ASCs were used for HUVEC treatment. ASCs were treated with differentially activated MQ-CM for 72 h as described above. After 72 h of treatment, ASCs were cultured in EBM for 24 h and CM were harvested. HUVECs were treated with MQ-treated ASC-CM for 24 h before beginning the angiogenesis assay. For this assay, HUVECs were plated on µ-slides (Ibidi, Germany) previously coated with Cultrex® Basement Membrane Extract (Bio-Techne, Ireland) according to the manufacturer’s instructions at a density of 7500 cells/50 µl with each treatment in triplicates. Phase contrast imaging was performed 6 h after plating using a CELENA S microscope (Logos Biosystems, South Korea), and images were analyzed using ImageJ Angiogenesis Analyzer software [27].

### Immunoblotting

For immunoblotting, protein lysates were obtained by direct addition of Laemmli buffer to 5×10^4^ to 2×10^5^ cells, followed by boiling at 75°C for 5 min, sonication, and addition of 10% Dithiothreitol (DTT) (Sigma-Aldrich, Austria). Protein samples were size-fractionated on pre-stained gradient polyacrylamide gels (Mini-PROTEAN®TGX Stain-Free™ Precast Gels, Biorad, Germany), blotted onto 0.2 µm PVDF membranes and blocked for 2 h in 5% reconstituted low-fat milk powder. After blocking, primary antibodies against hexokinase 1 (HK1), hexokinase 2 (HK2), PFKP, GAPDH, PKM2, LDHA, phosphorylated AKT (pAKT), AKT, phosphorylated p38 (p-p38), p38, phosphorylated signal-regulated kinase (pERK), ERK, phosphorylated mTOR, mTOR, phosphorylated focal adhesion kinase (pFAK), FAK (all from Cell Signaling Technology, The Netherlands), alpha-smooth muscle actin (SMA) (Dako, Denmark), integrin alpha 5 (ITGA5) (BD Biosciences, Germany), and integrin alpha V (ITGAV) (BD Biosciences, Germany) were applied and incubated overnight at 4°C. The membranes were then washed extensively and incubated with horseradish peroxidase-conjugated sheep anti-mouse and sheep anti-rabbit antibodies (all from Cell Signaling Technology, The Netherlands) for 1 h. Enhanced chemiluminescence reagent ECL (Bio-Rad, Germany) was used for visualization and reaction detection was performed using a Bio-Rad ChemidocMP gel analyzer (Bio-Rad, Germany). ImageLab software (version 5.2.1, Bio-Rad Laboratories, Germany) was used for gel quantification according to the user’s manual. Total protein loading was used as an internal loading control.

### RNA isolation and quantitative RT-PCR

RNA isolation was performed using TRI-Reagent (Sigma-Aldrich, Austria), and cDNA synthesis was performed using random hexamer primers and iScript cDNA-Synthesis kit (Bio-Rad, Germany) as previously described [24]. The SsoAdvanced™ Universal SYBR® Green Supermix Kit (Bio-Rad, Germany) was used in duplicates for quantitative PCR reactions on a CFX96-qPCR machine (Bio-Rad, Germany) according to the following protocol: one cycle at 95°C for 2 min, followed by 40 cycles at 95°C for 15 s, 60°C for 15 s, and 72°C for 10 s. Gene expression analysis was performed using CFX Manager software (version 3.1, Bio-Rad, Germany). CT values were normalized to the mean expression value of the reference gene 18sRNA and are presented as -ΔCT. All primers used in this study were designed using NCBI Primer-BLAST software (www.ncbi.nlm.nih.gov/tools/primer-blast) and synthesized by Microsynth Austria. Specificity was tested by evaluating the melting curve. Primer pairs are listed in Supplement Table 2.

### Statistics

Experiments were independently repeated at least three times with cells from different donors. Descriptive statistics were used to assess data quality. The Kolmogorov-Smirnov test was used to assess the Gaussian distribution. Data analysis was performed using unpaired Student’s *t*–test, Mann-Whitney *U* test, non-parametric Kruskal-Wallis test, and one-way ANOVA test combined with Dunnett’s test for multiple comparisons. Data are presented as mean ± and standard error of the mean (SEM). *P* values <0.05 were considered statistically significant. Prism 8 (GraphPad PRISM Software Inc., version 8.0) was used for statistical analysis. ImageJ (1.46r, Java 1.6.0_20, USA) was used for quantification of microscopic images.

## Results

### CD34^+^ preadipocytes are present in human granulation tissue

**Fig. 1:**
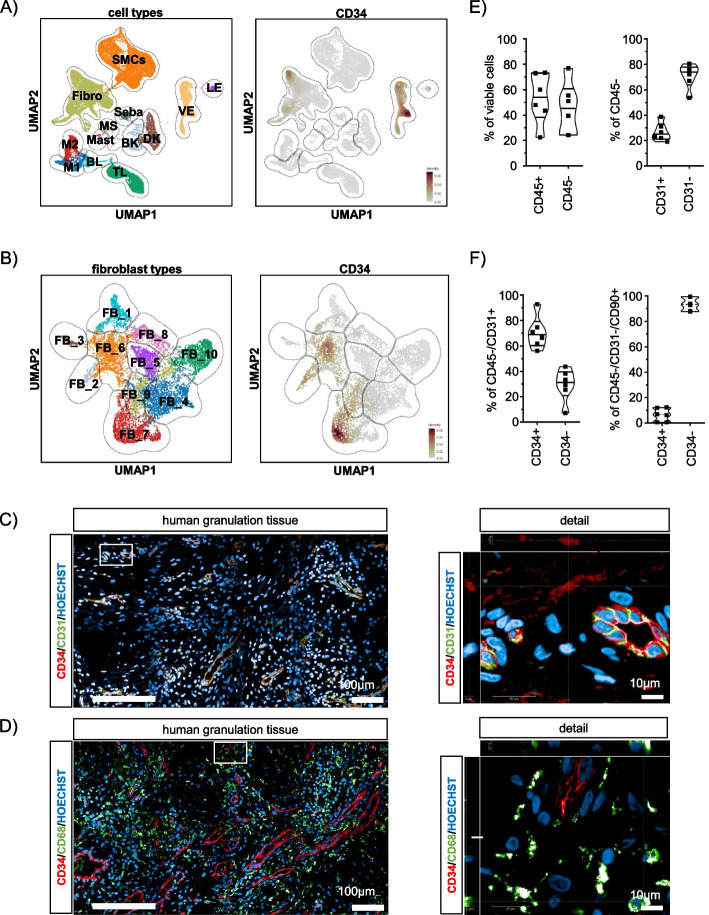
CD34^+^/CD31^−^ cells are present in human wound granulation tissue. **A** Overview of human granulation tissue cellularity and identification of CD34-expressing cells shown as uniform manifold approximation and projection (UMAP) plots. **B** Unsupervised clustering of fibroblast subtypes and expression of CD34. **C** Immunofluorescence staining of a representative granulation tissue section from a chronic lower limb wound in a 53-year-old male patient. The higher magnification detail (right image) indicates the presence of CD34^+^/CD31^−^ single and CD34^+^/CD31^+^ double positive cells. **D** Immunofluorescence staining of the same specimen. The higher magnification detail (right image) shows the proximity of CD68^+^ cells to CD34^+^ cells. **E**, **F** Flow cytometry analysis of single-cell suspensions from human granulation tissue samples (*n *= 6). **E** Left panel: Frequency of CD45^+^ and CD45^−^ cells shown as a percentage of viable granulation tissue cells. Right panel: Frequency of CD31^+^ cells shown as a percentage of CD45^−^ cells. **F** Left panel: frequency of CD34^+^ endothelial progenitor cells shown as a percentage of CD45^−^/CD31^+^ endothelial cells. Right panel: frequency of CD34^+^ cells shown as a percentage of CD45^−^/CD31^−^/CD90^+^ cells. Details of patients are described in Supplement Table 1

Lineage tracing experiments in mice suggested the existence of a CD34^+^/CD29^+^/SCA1^+^ adipocyte precursor cell population in the wound tissue of acute wounds [22]. This cell population exhibited a fibrotic rather than adipogenic phenotype and its presence was dependent on the infiltration of wound tissue by CD301b^+^ macrophages [22]. However, a corresponding CD34^+^ cell population has not yet been described in human wounds. Therefore, we re-analyzed publicly available single-cell RNA sequencing data derived from the human wound tissue of eleven patients with diabetic foot ulcers [18]. The dataset contained 18891 cells from seven patients with healing ulcers and 11839 cells from four patients with non-healing ulcers. Unsupervised clustering identified the following main cell clusters: smooth muscle cells (SMCs), fibroblasts (Fibro), endothelial cells (vascular endothelial (VE), lymphatic endothelial (LE)), keratinocytes (basal keratinocytes (BK) and differentiated keratinocytes (DK)), sebaceous gland cells (Seba), melanocytes and Schwann cells (MS), and immune cells (macrophages (M1 and M2), lymphocytes (B-lymphocytes (BL) and T-lymphocytes (TL)), and mast cells (MAST)) (Fig. 1A, left panel)). CD34 expression was detected in the vascular endothelial cell (VE) cluster and in parts of the fibroblast (Fibro) cluster (Fig. 1A, right panel). Subclustering of fibroblast subpopulations in diabetic foot ulcer samples revealed that 3 out of 10 fibroblast subclusters were consistently CD34-positive (Fig. 1B). Of note, the fibroblast clusters FB_3, FB_4, and FB_5, which were described by Theocharidis et al. [18] to correlate with wound healing in the original study were all CD34-negative, whereas the fibroblast cluster associated with impaired wound healing (FB_2) showed CD34 expression (Fig. 1B and Supplement Fig. 1). Overall re-analysis of single-cell RNA sequencing data revealed that cells from CD34^+^ fibroblast clusters were more frequent in samples from patients with impaired wound healing and that these clusters also showed higher CD34 expression (Supplement Fig. 1).

To further demonstrate the presence of CD34^+^/CD31^−^ preadipocytes in human wound tissue, we applied multiplex immunohistochemistry and immunofluorescence staining to human granulation tissue specimens. Whole-mount imaging revealed a distinct pattern of CD34^+^/CD31^−^ cells within the granulation tissue (Supplement Fig. 2). Areas of CD34^+^/CD31^−^ cells dominated the basal regions of both granulation tissue and adjacent unwounded tissue, whereas CD34^+^/CD31^+^ cells were associated with the vasculature in the apical regions of remodeling wound tissue. Detailed imaging using immunofluorescence-labeled antibodies and confocal microscopy revealed localization of CD34^+^/CD31^−^ preadipocytes in the intervascular space and localization of CD34^+^/CD31^+^ endothelial progenitor cells almost exclusively at the basement membrane of wound bed capillaries (Fig. 1C). Consistent with observations in murine wounds [22], we observed CD34^+^ cells in human granulation tissue near to clusters of CD68^+^ macrophages (Fig. 1D). Using an antibody marker panel applicable to the detection of adipose-derived stem cells (ASCs) in subcutaneous adipose tissue [28] (Supplement Fig. 3), multichannel flow cytometry analysis applied to single cell fractions isolated from human granulation tissue samples from chronic wounds also confirmed the presence of preadipocytes in human wound tissue. We found that CD45^−^/CD31^−^/CD90^+^/CD34^+^ preadipocytes comprised a population of about 6.5% (mean ± SD, 6.5% ± 5.2) of CD45^−^/CD31^−^/CD90^+^ cells (Fig. 1F, right panel), corresponding to approximately 1.5% (1.6% ± 1.2) of total viable human granulation tissue cells (Supplement Fig. 4). Other cell populations included those expressing the pan-hematopoietic marker CD45 (53.3% ± 19.6, Fig. 1E, left panel), while 45.2% ± 20.22 were non-hematopoietic CD45^−^ cells. Approximately a quarter (1/4) of the non-hematopoietic CD45^−^ cells were positive for the endothelial cell marker CD31 (CD45^−^/CD31^+^ endothelial cells: 26.8% ± 7.1) (Fig. 1E, right panel), corresponding to about 10% (11.4% ± 4.2) of all viable granulation tissue cells (Supplement Fig. 4). Approximately 70% of the CD45^−^/CD31^+^ cells co-expressed CD34 (70.5% ± 12.7) (Fig. 1F, left panel), supporting our observations by immunofluorescence staining. These results suggest that CD34^+^/CD31^−^ preadipocytes are present in human chronic wound granulation tissue.

**Fig. 2:**
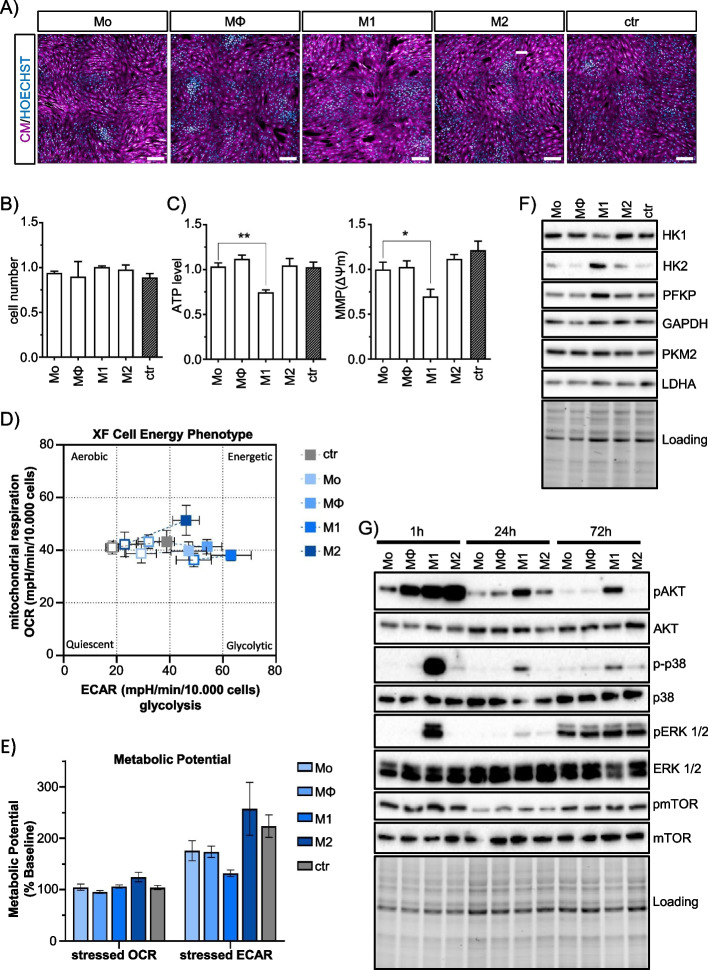
M1 macrophage secretome effects on human ASCs metabolism. **A** Representative confocal images of human ASCs cultured in differentially activated MQ-CM for 72 h. Cells were stained with CellMask Orange to visualize cell morphology. Scale bars indicate 200 µm. **B** Quantification of relative cell numbers in 72 h treated ASC. **C** ATP levels measured by ATP-Red and mitochondrial membrane potential (MMP) determined by TMRM staining (*n *= 6). Asterisks indicate *p* values of *p*<0.05 (*) and *p*<0.01 (**). **D** Energy profiling of MQ-CM treated ASCs at 72 h determined by Agilent Seahorse technology. The graph shows the correlation of mitochondrial respiration determined by oxygen consumption rate (OCR) and glycolysis measured by extracellular acidification rate (ECAR). Open squares indicate levels under basal conditions; closed squares indicate levels under oligomycin-induced mitochondrial uncoupling (stressed conditions) (*n *= 4). **E** Metabolic potential indicates the ability of cells to meet an energy demand via mitochondrial respiration and glycolysis. This is shown as the percentage increase of stressed OCR over baseline OCR and stressed ECAR over baseline ECAR (*n *= 4). **F** Representative immunoblots of MQ-CM treated ASC at 72 h analyzed for the expression of key regulatory enzymes of glycolysis. **G** Representative immunoblots of MQ-CM-treated ASCs showing levels and activation of intracellular kinases over time. All data are shown as mean ± SEM

**Fig. 3:**
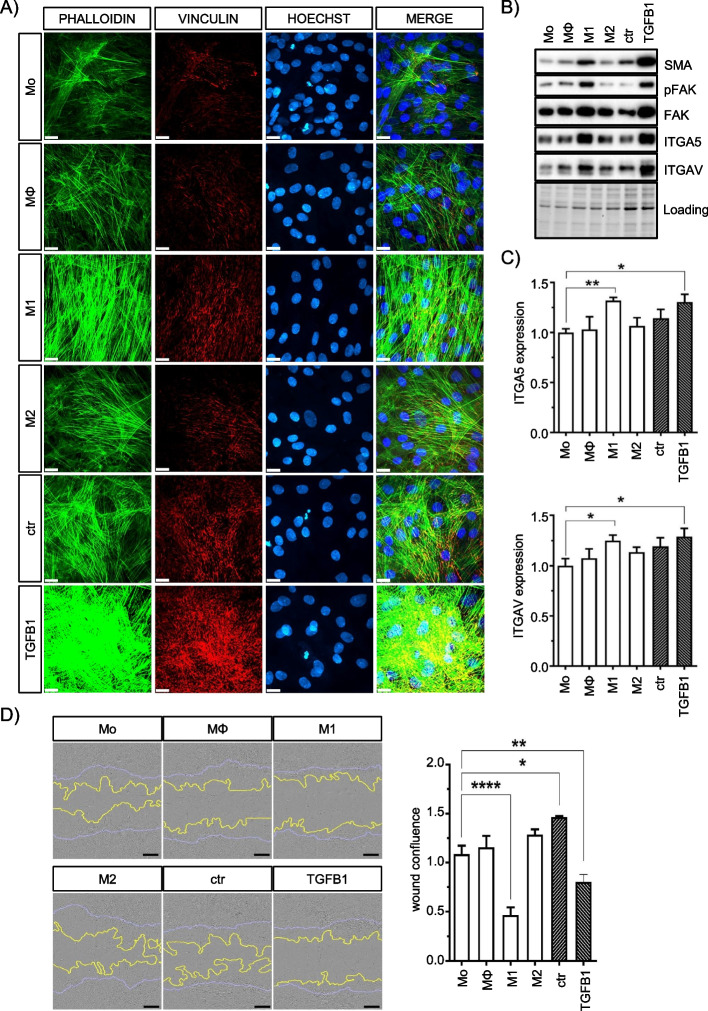
M1 macrophage secretome induces stress fiber formation in ASCs. **A** Representative confocal images of MQ-CM-treated ASCs at 72 h showing increased actin stress fibers (phalloidin) and focal adhesions (vinculin) in MQ-CM- and TGFB1-treated ASCs. Scale bars indicate 20 µm. **B** Representative immunoblots of MQ-CM-treated ASCs after 72 h analyzing levels of ECM-cytoskeleton signaling enzymes including SMA, phosphorylation of FAK and RGD-recognizing integrins ITGA5 and ITGAV. **C** Flow cytometry analysis of cell surface expression of ITGA5 and ITGAV (*n *= 6). **D** Representative images of a conventional scratch assay showing the migration ability of MQ-CM-treated ASCs. The blue lines indicate the initial scratch lines; the yellow lines indicate the relative migration distance after 24 h. Scale bars indicate 200 µm. Quantification of the relative migration distance was calculated using INCUCYTE^TM^ software based on cell confluence within the wound region over time (*n *= 4). All data are shown as mean ± SEM. Asterisks indicate *p* values of *p*<0.05 (*), *p*<0.01 (**), and *p*<0.0001 (****). TGFB1 treatment served as a positive control for maximal stress fiber induction

**Fig. 4:**
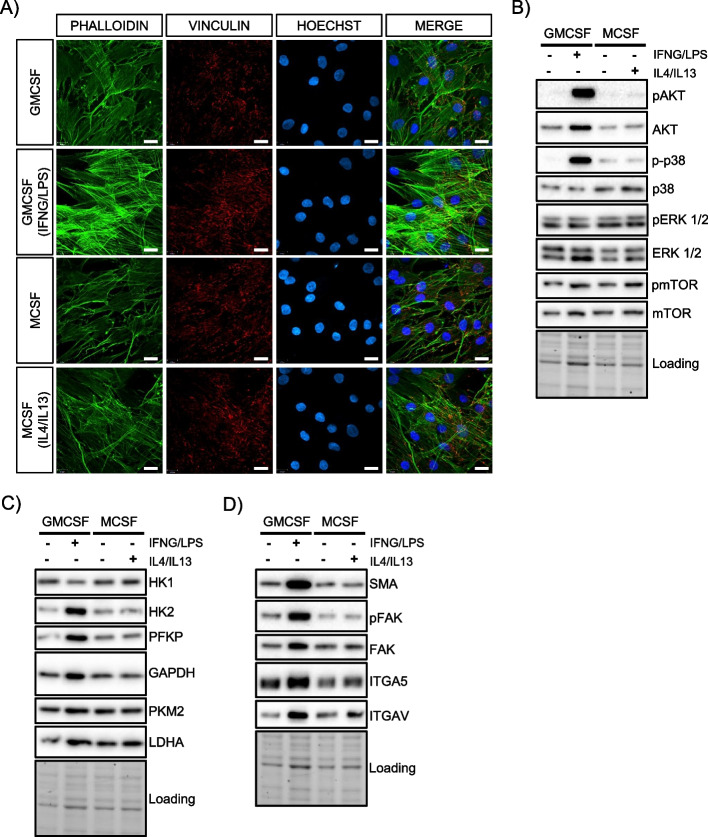
Monocyte-derived macrophages prove M1 effects on ASCs. **A** Representative confocal images of stress fibers (phalloidin) and focal adhesions (vinculin) of ASCs exposed to CM of CD14^+^ monocyte-derived pro-inflammatory (GMCSF and GMCSF (IFNG/LPS)) or anti-inflammatory (MCSF and MCSF (IL4/IL13)) macrophages. Scale bars indicate 20 µm. **B**–**D** Representative immunoblots analyzing the levels of **B** intracellular kinases, **C** key enzymes regulating glycolysis, and **D** ECM-cytoskeleton signaling

### M1 macrophage secretome stimulates ASC glycolysis

During wound healing, cells of different origins continuously influence the phenotype of neighboring cells through paracrine signaling and extracellular matrix (ECM) remodeling [3, 10]. To cope with changing environmental signals, cells adapt their physiology by modifying cellular metabolism, mitochondrial activity, and cytoskeletal structure [29, 30]. Based on the observation that CD34^+^/CD31^−^ preadipocytes localize close to CD68^+^ macrophages in human granulation tissue (Fig. 1D), we aimed to mimic this interaction in vitro by exposing primary human ASCs isolated from subcutaneous adipose tissue to a conditioned medium (CM) of distinct polarized macrophages.

Using our published human macrophage polarization model [23], we generated CM from quiescent macrophages (MΦ), IFNG/LPS-activated, pro-inflammatory M1 macrophages, IL4/IL13-activated, anti-inflammatory M2 macrophages, and monocytes (Mo) as control and cultured ASCs in these media up to three days. Except for an increase in cell size of M2-CM treated cells, MQ-CM treatment did not induce any obvious changes in cell morphology, proliferation, or viability (Fig. 2A, B, and Supplement Fig. 5). However, an in-depth analysis of cellular metabolism revealed a decrease in mitochondrial activity. A significant reduction in ATP levels and mitochondrial membrane potential (MMP) was observed in M1-CM treated cells using the live cell fluorescence imaging probe ATP-Red and the lipophilic cation tetramethylrhodamine methyl ester TMRM, respectively (Fig. 2C).

**Fig. 5:**
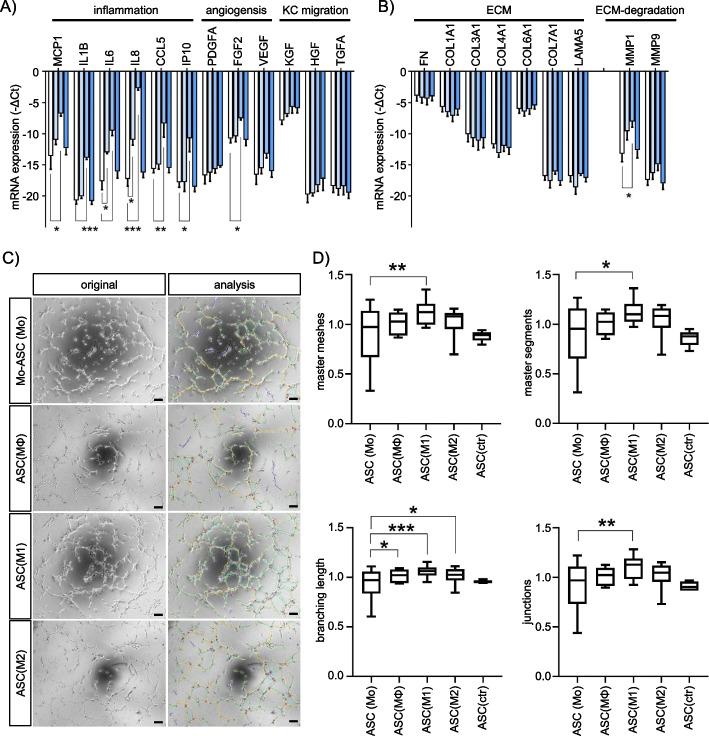
M1 macrophage secretome induces a myofibroblast-like secretory phenotype in ASCs. **A**, **B** Gene profiling by quantitative qPCR mRNA analysis of MQ-CM-treated ASCs at 72 h. Genes analyzed were clustered for **A** potential inflammatory, angiogenic, and keratinocyte (KC) mobilizing function (*n *= 3) or **B** ECM remodeling properties (*n *= 5). Data are shown as -ΔCt ± SEM. **C** Representative images of HUVEC-based in vitro angiogenesis assay and analysis thereof at 6 h. **D** Quantification of master meshes, master segments, branch lengths, and junctions of identified HUVEC structures. Data are shown as mean ± SEM (*n *= 6). Asterisks indicate *p* values of *p*<0.05 (*), *p*<0.01 (**), and *p*<0.001(***)

The reduced cellular energy production could be indicative of a metabolic shift from oxidative phosphorylation (OXPHOS)-dependent ATP production to aerobic glycolysis, a pathway less efficient for ATP production and known as the Warburg effect in cancer cells [31–33].

Therefore, we performed an automated measurement of energy metabolism based on Agilent’s Seahorse technology (Fig. 2D) [34, 35]. We calculated the cellular oxygen consumption rate (OCR), a measure of mitochondrial respiration based on oxygen concentrations, and the extracellular acidification rate (ECAR), which directly correlates with the extent of glycolysis. Under basal conditions, OCR and ECAR were significantly decreased in M1-CM-treated cells (Fig. 2D, open squares), which correlated with decreased ATP levels and reduced mitochondrial membrane potential. Challenging cells with oligomycin, a compound that inhibits mitochondrial ATP production and forces cells into a glycolytic metabolism [36], revealed a limited metabolic flexibility of M1-CM treated cells (Fig. 2E). This data strongly suggests that M1-CM treatment of ASCs induces a metabolic shift from OXPHOS-dominated to glycolysis-based metabolism.

To investigate whether the observed metabolic changes also correlated with altered protein expression of key enzymes regulating glycolysis, protein levels were determined by immunoblotting for hexokinase 1 and 2 (HK1 and HK2), phosphofructokinase platelet isoform (PFKP), glyceraldehyde-3-phosphate dehydrogenase (GAPDH), pyruvate kinase M2 (PKM2), and lactate dehydrogenase (LDHA) (Fig. 2F). While levels of HK1, which regulates the first step of glycolysis by phosphorylating glucose to glucose-6-phosphate [37, 38], were decreased in M1-CM-treated cells, the second HK-isoform, HK2, was increased. In addition, levels of the ubiquitously expressed PFK-isoform PFKP, which catalyzes the irreversible conversion of fructose-6-phosphate and ATP to fructose-1,6-biphosphate [39], were also increased in M1-CM-treated cells. Interestingly, the downstream glycolytic enzymes GAPDH, PKM2, and LDHA were not regulated at the protein level. From this data, we concluded that M1-CM constituents affected the early steps of glucose metabolism and shifted cells into increased glycolytic activity with decreased ATP production.

### M1 macrophage secretome activates AKT, p38-MAPK, and ERK1/2 kinases in ASCs

Previous studies on cancer cell lines have shown that metabolic changes consequently affect intracellular kinase cascades [40]. These kinases, such as the serine/threonine kinase AKT/PKB, which is activated downstream of class I phosphatidylinositol 3-kinase (PI3K) upon RTK/GPCR stimulation, are known to affect metabolic cascades by phosphorylation of key enzymes regulating glycolysis and OXPHOS [41]. Since phosphorylation of kinases is a rapid process, we performed time-course experiments to analyze early and late events in kinase activation in ASCs exposed to MQ-CM (Fig. 2G). MQ-CM generally induced phosphorylation of AKT/PKB at Ser437 in ASCs at an early time point (1 h) of treatment. However, the phosphorylation persisted only in M1-CM-treated cells. A similar response was observed for the mitogen-activated protein kinase (MAPK) p38-MAPK, which is characterized as a “stress-responsive” MAPK subfamily [42, 43]. p38-MAPK was phosphorylated at Thr180/Tyr182 in M1-CM-treated cells, and p-p38-MAPK levels greatly increased at 1 h followed by a subsequent decrease with time of exposure. In contrast, p44/42-MAPK (ERK1/2), which is predominantly activated by mitogens and growth factors through the RAF-MEK1/2 pathway [44], was exclusively phosphorylated in M1-CM treated cells after 1 h. No differences in phosphorylation were observed after 72 h of treatment. Phosphorylation of the mammalian target of rapamycin (mTOR) at Ser2448, which acts as a nutrient sensor regulating cell growth and is a known target of activated AKT/PKB, was not affected by different MQ-CM treatments. In conclusion, the data suggest that M1-CM, but not other MQ-CM, induces stress-associated kinase cascades in ASCs that are likely to affect other cellular properties and structures.

### M1 macrophage secretome induces stress fiber formation and reduces ASC motility

Assuming that changes in subcellular structures result from decreased metabolic activity and induction of potential stress kinase pathways, we analyzed cytoskeletal remodeling and cell surface markers of MQ-CM-treated ASCs. Although we did not detect obvious changes in cell morphology by plasma membrane staining (Fig. 2A), visualization of cytoskeletal actin filaments, and cell surface focal adhesions (FA) by phalloidin and vinculin staining [45] showed dramatically increased stress fiber formation and matrix adhesion in M1-CM-treated ASCs (Fig. 3A). Actin stress fibers were prominent in M1-CM-treated ASCs, whereas actin bundles were consistently thinner and less organized in other MQ-CM treatments. Since recent studies in mice have demonstrated the potential of ASCs to differentiate into a myofibroblast-like phenotype [22], we treated ASCs with high concentrations (10 ng/ml) of recombinant TGFB1 to induce maximal stress fiber induction. As expected, the extent of stress fiber assembly exceeded even that obtained by M1-CM treatment, thus providing a positive control for maximal myofibroblast-like differentiation potential of ASCs. At the molecular level, actin alpha 2 (ACTA2), an actin isoform associated with TGFB1-mediated stress fiber assembly and also referred to as SMA [39], was strongly induced in M1-CM- and TGFB1-treated cells. Similarly, the non-receptor tyrosine kinase focal adhesion kinase (FAK), which localizes to focal adhesion sites upon activation, was also strongly induced. FAK activation is initiated by autophosphorylation of Tyr397 at the FERM-domain linkage region, which subsequently allows full activation by SRC kinase-dependent phosphorylation at tyrosine in the kinase domain [46, 47]. Both, basal FAK and Tyr397 phosphorylation levels were increased in M1-CM-treated cells (Fig. 3B). This coincided with increased levels of integrin alpha 5 (ITGA5) and integrin alpha V (ITGAV), two Arg-Gly-Asp (RGD) motifs recognizing integrins involved in the maintenance of ASC stemness maintenance [24, 48], indicating an increased matrix-cell interaction in M1-CM-treated cells (Fig. 3B and C). In addition, molecular data correlated with functional cell characteristics using a multi-well scratch wound assay. Reduced cell motility was observed only in M1-CM and TGFB1-treated ASCs (Fig. 3D), supporting our findings of pronounced actin cytoskeleton assembly with increased cell surface focal adhesions.

### CD14^+^ monocyte-derived macrophages confirm effects of M1-CM on ASCs

To confirm our observations on the molecular regulation of kinases and glycolytic effects of IFNG/LPS-activated, THP1-derived macrophage-CM (M1-CM) on ASCs, we used a second in vitro model based on human-derived monocytes to generate distinct macrophage subtypes. We isolated CD14^+^ monocytes from human peripheral blood mononuclear cells (PBMC) and generated CM from GMCSF ± IFNG/LPS (pro-inflammatory M1 macrophages) or MCSF ± IL4/IL13 (anti-inflammatory M2 macrophages) stimulated CD14^+^ monocytes. Experimental results using these CM on ASCs confirmed all the regulations induced by THP1-derived macrophages, including increased actin stress fiber formation, elevated levels of HK2 and PFKP, and altered regulation of intracellular kinases and cell adhesion proteins (Fig. 4A–D).

### M1 macrophage secretome alters angiogenic properties of ASCs

Our data suggest that under inflammatory conditions, ASCs adopt a myofibroblast-like phenotype characterized by decreased metabolic activity, increased stress fiber assembly, and decreased cell motility. To decipher whether the M1-CM-induced ASC phenotype exhibits additional features of a secretory myofibroblast-like phenotype, we analyzed the mRNA levels of cytokines, ECM-substrates, and matrix remodeling enzymes associated with inflammation, angiogenesis, and keratinocyte (KC) migration (Fig. 5A and B). M1-CM induced the expression of the pro-inflammatory cytokines MCP1, IL1B, IL6, IL8, and CCL5 (RANTES) as well as the angiogenesis regulating cytokine FGF2 (Fig. 5A). With the exception of an increase in the ECM degrading protease MMP1 under M1-CM treatment, none of the investigated ECM components were significantly upregulated or downregulated at the mRNA level. These combined observations suggested M1-CM treated ASCs to adopt an oxidative stress-induced senescence-associated secretory phenotype (SASP) [49] under inflammatory conditions characterized by reduced metabolism, a myofibroblast-like phenotype, and expression of pro-inflammatory cytokines.

To investigate the functional consequences of the observed mRNA changes on intercellular signaling, we performed an angiogenesis assay. Human umbilical vein endothelial cells (HUVECs) were exposed to differential ASC-CM during tube formation. ASCs were pre-exposed to differential MQ-CM for 72 h before harvesting CM for HUVEC treatment. Analysis of the developed HUVEC network units revealed an increased assembly of meshes, segments, branch length, and junctions for HUVECs treated with CM from pro-fibrotic, M1-CM-treated ASCs (Fig. 5C and D). Thus, somewhat unexpected, our data suggested that the M1-induced, pro-fibrotic ASCs promoted angiogenesis compared to ASCs treated with any other MQ-CM.

### Modulation of IL1B and TGFB1 signaling attenuates stress fiber formation in ASCs

**Fig. 6:**
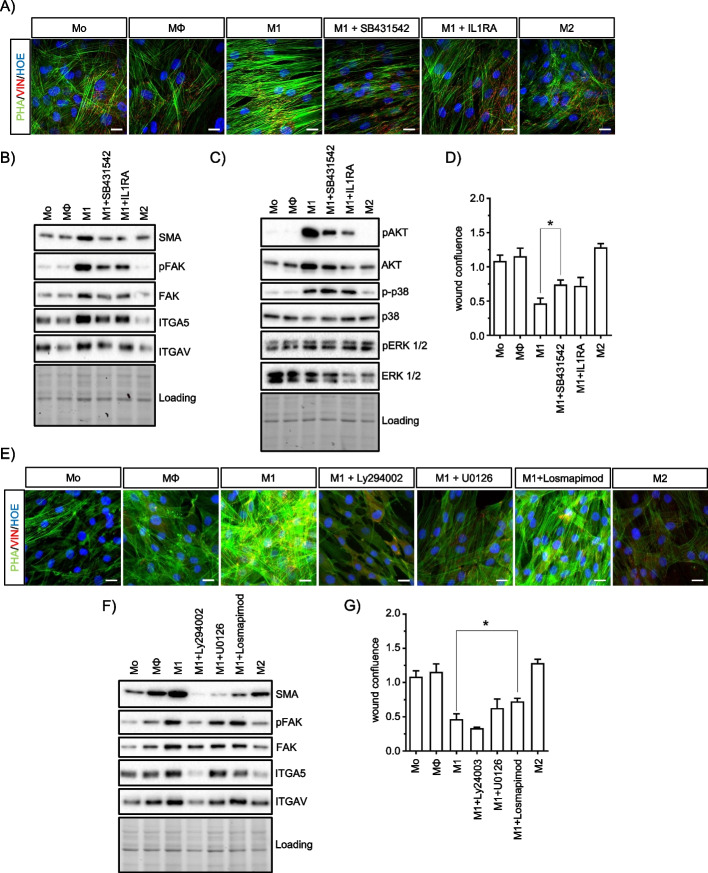
Stress fiber assembly in M1-CM-treated cells is mediated by IL1B, TGFB1, and AKT signaling. **A** Representative confocal microscopy images of MQ-CM-treated ASCs at 72 h. Supplementation of M1-CM with TGFB1 inhibitor SB431542 or IL1B inhibitor IL1RA reduced stress fiber assembly. **B**, **C** Representative immunoblots analyzing ECM-cytoskeletal signaling (**B**) and intracellular activated kinases (**C**). **D** Quantification of cell migration of MQ-CM-treated ASC with at least partially rescued by impaired IL1B and TGFB1 signaling. **E** Representative confocal microscopy images of MQ-CM-treated ASCs including co-treatment with either PI3K/AKT inhibitor LY294002 (25 µM), ERK1/2 inhibitor U0126 (10 µM), or p38-MAPK inhibitor Losmapimod (25 µM) during incubation with M1-CM treatment. **F** Representative immunoblots analyzing ECM-cytoskeleton signaling. **G** Cell migration of MQ-CM-treated ASCs with impaired AKT, ERK1/2, or p38-MAPK signaling. All scale bars indicate 20 µm. All data are shown as mean ± SEM. Asterisks indicate *p* values of <0.05 (*)

Our previously published cytokine analysis of MQ-CM showed that IFNG/LPS-stimulated macrophages expressed high levels of IL1B and TGFB1 [23]. To evaluate the importance of these cytokines in the development of a pro-fibrotic ASC phenotype, we supplemented pro-inflammatory M1-CM with either the IL1 receptor antagonist IL1RA or the TGFB1 receptor kinase inhibitor SB431542. Inhibition of both IL1B and TGFB1 significantly reduced actin stress fiber formation in M1-CM-treated cells and partially reversed the induction of SMA, FAK, ITGAV, and ITGA5 (Fig. 6A and B). In addition, the phosphorylation of AKT was reduced compared to p38 and ERK (Fig. 6C). Similarly, cell motility was moderately restored upon inhibition of IL1B and TGFB1 signaling (Fig. 6D). To distinguish differences in IL1B and TGFB1 signaling, ASCs were treated with either recombinant human IL1B or TGFB1. Treatment of ASCs with either cytokine increased the number of stress fibers and decreased cell motility (Supplement Fig. 6A-E). At the molecular level, IL1B and TGFB1 upregulated SMA, p-FAK (Tyr397), and ITGA5, suggesting that these cytokines mediate, at least in part, the M1-CM induced pro-fibrotic effects in ASCs (Supplement Fig. 6C). Although both IL1B and TGFB1 induced similar phenotypes, phosphorylation of AKT and p38-MAPK was higher in IL1B-treated cells, suggesting a greater activation of cytokine-specific cellular response pathways by IL1 receptor signaling (Supplement Fig. 6D).

### Effects of M1 macrophage secretome on ASCs are mediated by phosphorylation of AKT at Ser437

We also assessed the importance of downstream kinase activation in macrophage-induced pro-fibrotic ASCs by inhibiting either the PI3K/AKT pathway with LY294002, ERK1/2 signaling with U0126, or p38-MAPK signaling with losmapimod (Fig. 6E–G, Supplement Fig. 7A, B). Inhibition of AKT/PKB phosphorylation, but not other kinases, was sufficient to reduce stress fiber assembly in M1-CM-treated ASCs. Characteristically, in addition to reduced stress fibers, these cells exhibited reduced SMA, ITGAV, ITGA5, and decreased phosphorylation of FAK. Our data suggest a regulatory function of AKT/PKB in actin cytoskeleton remodeling, as previously described [50]. In conclusion, our data suggest that actin stress fiber assembly under M1-CM depends on PI3K-dependent AKT phosphorylation at Ser437 rather than on activation of ERK1/2 or p38-MAPK, and is mediated, at least in part, by IL1B and TGFB1.

## Discussion

### CD34^*+*^ cells are present in human granulation tissue

Human skin wound healing resembles the perfect interplay of numerous different cell types [1]. While classic cellular key players include macrophages, fibroblasts, and endothelial cells [3], recent research has identified adipocytes as essential participants [51, 52]. Whether adipocytes repopulate from wound-infiltrating adipose-derived stem cells (ASCs) or differentiate from another regenerative cell source remains elusive. In this study, we identified CD45^−^/CD31^−^/CD90^+^/CD34^+^ cells in human granulation tissue gained from chronic wounds. These cells represented approximately 1.5% of all nucleated wound cells and were generally found at the base of the granulation tissue and in close proximity to CD68^+^ macrophages. We speculate that these CD45^−^/CD31^−^/CD90^+^/CD34^+^ cells may represent a subpopulation of cells corresponding to CD34^+^/CD29^+^ adipocyte precursor cells previously found in murine wounds [22]. In addition, re-analysis of recently published single-cell RNA sequencing data identified two major populations of CD34^+^ cells in human chronic wound granulation tissue. While the identified CD34^+^/CD31^+^ cells were attributed to endothelial progenitor cells, CD34 was also expressed within two fibroblast subpopulations [18]. We speculate that these cells may still have adipogenic potential as our previous observations found a small number of granulation tissue-resident cells capable of adipocyte differentiation [23].

### The fate of CD34^*+*^ cells in a wound environment

Based on our observation that CD34^+^ cells localize in close proximity to CD68^+^ macrophages in vivo, we hypothesized that a mutual crosstalk between CD34^+^ cells and macrophages through shared extracellular matrix (ECM) components is likely. In this study, we used CD68 as a marker for granulation tissue macrophages. It should be noted that CD68 is not exclusively expressed in monocytes/macrophages, but has also been reported to be expressed in non-myeloid and non-hematopoietic cells [53]. However, if strong CD68 expression is used as an indicator of highly phagocytic cells to define inflammatory areas in granulation tissue, this staining provides valuable information about the microenvironment of CD34^+^ cells. We aimed to mimic these microenvironmental cues to which CD34^+^ cells might be exposed within wound tissue by using primary ASCs isolated from human subcutaneous adipose tissue as an in vitro model for granulation tissue-resident CD34^+^ cells and exposing these ASCs to secretomes of differentially activated macrophages corresponding to different stages of wound healing.

In our model, IFNG/LPS stimulation of macrophages replicates the early phase of wound healing when macrophages primarily focus on phagocytosis of microbes, accompanied by robust inflammatory signaling initiated by first-line neutrophils [54]. This phagocytic macrophage phenotype closely resembles the initial-phase macrophages encountered in physiological wound healing by days 2-5 [55]. If this phenotype persists, it can become a hallmark of chronic wounds, where ongoing cell death and microbial invasion impede the healing process [56]. In acute wounds, the influence of these macrophages on regenerative cells is transient, mainly limited to the initial inflammatory phase and diminishes as wound healing progresses. This transition is critical for triggering the initial stages of vascularization and extracellular matrix deposition.

Conversely, IL4/IL13 stimulation of macrophages simulates the anti-inflammatory signaling that characterizes the proliferative phase of acute wounds, which is largely mediated by M2a-like macrophages in vivo. Interestingly, these M2 macrophages induce an anti-inflammatory ASC phenotype with a limited ability to induce tube formation in HUVEC cells. However, they may provide an anti-fibrotic cell phenotype capable of differentiating into various types of scar tissue cells. This crosstalk between IL4/IL13-stimulated macrophages and ASCs predominantly occurs in wounds inclined to heal during the proliferative phase by days 5–7 [55], setting it apart from the interactions in chronic wounds.

It is crucial to note that our model provides a simplified representation of the intricate regulatory microenvironment present in wounds. In vivo, granulation tissue formation is significantly shaped by macrophage subtypes, which dynamically adapt their cellular responses to the presence of DAMPs and PAMPs [57, 58]. This adaptability results in variations in both the number and phenotype of macrophages in chronic and acute wounds [59–61]. Notably, chronic wound macrophages exhibit reduced efferocytosis [62, 63] and an increased propensity to transition into a senescence-associated secretory phenotype, coupled with reduced M2 polarization [64, 65]. Determining precisely which chronic or acute wound-induced macrophage subtype is replicated by our stimulation with IFNG/LPS or IL4/IL13 is a challenging task. However, it is worth noting that several cytokines produced in our macrophage model [23] resemble the profiles of M1 and M2 macrophage subtypes identified in human wounds by Theocharidis et al. [18]. These overlapping cytokine profiles encompass critical mediators such as IL1B, TNFA, IL6, and IL8 for M1 macrophages and CCL8, CCL2, IL13, and CCL23 for M2 macrophages, suggesting that our macrophage model successfully captures certain features of in vivo macrophages.

Keeping this limitation in mind, we demonstrated that conditioned media (CM) from pro-inflammatory macrophages (M1) induced a pro-fibrotic phenotype in ASCs. This phenotype was characterized by increased stress fiber assembly, decreased cell motility, and an elevated inflammatory cytokine profile. The pro-fibrotic determination of human preadipocytes by macrophages was previously described as increased ECM substrate deposition in preadipocytes exposed to CM from LPS-stimulated macrophages [66]. It has also been reported that preadipocytes adopt a myofibroblast-like phenotype in murine wound models [22]. Our presented data suggest that a pro-fibrotic phenotype is only induced in ASCs under inflammatory conditions by neighboring pro-inflammatory macrophages.

Quiescent (MΦ) or anti-inflammatory (M2) macrophages did not promote fibrotic differentiation of ASCs, which is consistent with considerations of in vivo wound remodeling and may explain the repopulation of adipocytes within granulation tissue [23]. In the present study, inhibition of the key inflammatory cytokines TGFB1 and IL1B partially rescued ASCs from undergoing fibrotic remodeling. However, the cells retained elevated levels of SMA, FAK, and integrins compared to anti-inflammatory MQ-CM-treated ASCs.

In contrast, ASCs treated with recombinant TGFB1 or IL1B exhibited a pro-fibrotic phenotype comparable to ASCs treated with pro-inflammatory MQ-CM. These results confirm TGFB1 and IL1B as cytokines with high fibrotic activity and activation of related downstream kinase pathways. In this regard, activation of the PI3K/AKT and the p38-MAPK pathway was established and maintained over time in cells treated with either M1-CM, TGFB1, or IL1B. Notably, IL1B signaling appeared to outperform TGFB1 effects in terms of both AKT/PKB and p38-MAPK phosphorylation. Both pathways are known to be sensitive to activation of G-protein-coupled receptors (GPCRs) and receptor tyrosine kinases (RTKs) [67], and their inhibition partially rescued the M1-induced phenotype. Activation of actin remodeling kinase substrates such as Girdin/APE via PI3K/AKT [50] or HspB1 via p38-MAPK [68] may link kinase activation to stress fiber assembly in M1-CM treated ASCs. However, our data support the hypothesis that the pro-fibrotic effects of macrophages on ASCs are mediated intracellularly through the PI3K/AKT pathway.

Furthermore, increased AKT activation may contribute to the glycolytic metabolic shift observed in M1-CM-treated ASCs. A link between AKT/PKB activation and glycolysis can be inferred from inverse HK1 and HK2 expression studies [69, 70]. Mitochondrial-bound hexokinases regulate the first step of glucose metabolism by catalyzing the phosphorylation of glucose to glucose-6-phosphate [37, 38]. The levels of HK isoforms and their localization to mitochondria are mediated by AKT/PKB [70]. While HK1 localizes to mitochondria independently of AKT/PKB phosphorylation, HK2 necessarily depends on AKT/PKB phosphorylation to bind to mitochondrial membranes [69]. Moreover, HK2 is known to be regulated by growth factors [71] or by activation of HIF1A upon cellular stress signaling [72]. Pro-inflammatory MQ-CM treatment, as well as treatment with recombinant IL1B, strongly induced HK2 at the protein level while simultaneously decreasing HK1 levels.

A second link between AKT/PKB activation and glycolysis may be indicated by increased PFKP levels. Phosphorylation of PFKP at S386 by activated AKT/PKB inhibits binding of the E3 ligase TRIM21, thereby preventing PFKP from subsequent polyubiquitination and degradation [41]. The elevated PFKP protein levels observed in M1-CM-treated cells may result from stabilized PFKP.

Interestingly, HK2 and PFKP levels are highly expressed in cancer cells [73, 74], which correlates with higher glycolysis rates [41]. This is known as the Warburg effect, a mechanism used by cells to overcome energetic demands under hypoxic conditions [75]. Like cancer cells, M1-CM-treated ASCs decreased cellular oxygen consumption with a concomitant increase in extracellular acidification rate, as indicated by a shift from OXPHOS-dependent ATP production to an aerobic glycolysis-dominated metabolism.

In summary, pro-inflammatory M1-CM induced a pro-fibrotic ASC phenotype with features of the oxidative stress-induced senescence-associated secretory phenotype (SASP) [49, 76, 77].

###  The possible role of granulation tissue residing CD34^+^ cells in the clinical setting

Although recent data describe the pro-healing effects of transplanted ASCs on chronic wounds [78], the clinical effects are moderate and hardly justify the cost and effort. By challenging the fate decision and adoption strategies of ASCs in an in vitro wound environment, we found that an inflammatory microenvironment induced pro-inflammatory cytokine production in ASC.

Notably, the cytokine secretome of ASCs induced by pro-inflammatory macrophages promoted pro-angiogenic effects in HUVECs, suggesting that this type of regenerative cell may be important for neo-angiogenesis in the wound bed and, thus, crucial for successful wound healing. The secretome of ASCs pretreated with anti-inflammatory MQ-CM did not induce these effects in HUVECs. Therefore, our data suggest that pro-inflammatory cytokine production by ASCs may, at least in part, promote neo-angiogenesis in wound healing.

### Supplementary Information

**Additional file 1:** **Supplement Figure 1.** CD34expression within cell clusters in human wound tissue. Expression of CD34 in fibroblast cell clusters and proportion of cells originating from diabetic foot ulcer (DFU) healers compared to non-healers for each cluster. Left panel: CD34 expression in CD34^-^ and CD34^+^ fibroblast cell clusters split by wound healing response. Right panel: Expression of CD34 in all identified fibroblast subpopulations showing high CD34 expression in three clusters (Fb_2, Fb_6, Fb_7). Data are shown as violin plots. **Supplement Figure 2.** CD34^+^ and CD31^+^ cells repopulate human granulation tissue. Immunohistochemistry of a representative section of granulation tissue from a chronic lower limb wound in a 53-year-old male patient stained for CD34 (pink) and CD31 (brown). Left image: Cross-sectional view of the granulation tissue specimen. Scale bar indicates 1 mm. Right image: Detailed view of the same specimen showing the distribution of CD34^+^ cells (black arrows). Scale bars indicate 100 µm. **Supplement Figure 3.** Flow cytometry gating strategy. Flow cytometry gating strategy that discriminates single cell populations to define CD45^-^/CD31^-^/CD90^+^/CD34^+^ adipocyte precursor cells. **Supplement Figure 4.** Flow cytometric analysis. Flow cytometric analysis of single cell suspensions from human granulation tissue samples (*n *= 6). Mean frequencies of cell types are shown as a percentage of total viable granulation tissue cells. **Supplement Figure 5.** Effect of MQ-CM on ASC size and viability. Data from ASCs cultured in differentially activated MQ-CM for 72 h are shown as fold change compared to monocyte-CM treatment (Mo) (*n *= 5). Cell volume and diameter were analyzed using CASY TT cell counter. Cell viability corresponds to the number of cells negative for Annexin V and 7AAD staining as assessed by flow cytometry. Data are shown as mean ± SEM. Asterisks indicate *p*-values of <0.01 (**). **Supplement Figure 6.** Comparison of IL1B- and TGFB1-mediated effects on ASC physiology. (A) Representative confocal microscopy images of ASCs cultured in the presence of recombinant human IL1B (10 ng/ml) or TGFB1 (10 ng/ml) for 72 h and analyzed for actin cytoskeleton remodeling. Scale bars indicate 20 µm. (B) Quantification of mitochondrial membrane potential (MMP) (*n *= 4). Data are shown as mean ± SEM. Asterisks indicate *p*-values of *p *< 0.01 (**). (C-E) Representative immunoblots of cell lysates of the respective cells analyzed for cell adhesion proteins (C), intracellular kinases (D), or glycolysis regulating enzymes (E). **Supplement Figure 7.** Efficacy of kinase inhibition. (A) Representative immunoblots of ASCs exposed to different MQ-CM for 1 h. Kinase inhibition of M1-CM treatment with kinase inhibitors Ly294001 (PI3K/AKT), U0126 (ERK1/2), and Losmapimod (p38-MAPK) demonstrated efficacy. (B) Representative microscopic images of phalloidin staining (green) for actin stress fibers in ASCs cultured in M1-CM supplemented with LY294001 (10 µM), U0126 (10 µM) or Losmapimod (10 µM) for 72 h.

**Additional file 2:** **Supplement Table 1.** Chronic wound patient characteristics. **Supplement Table 2.** Quantitative RT-PCR primers. All Primers used in this study are listed in the table below.

## Data Availability

All data supporting the conclusions of this article are available upon request at the corresponding author.
