# Supplementary material for: Preadipocytes in human granulation tissue: role in wound healing and response to macrophage polarization
Source: Inflamm Regen. 2023 Oct 31;43:53. doi: 10.1186/s41232-023-00302-5 (PMC10617061; doi:10.1186/s41232-023-00302-5)
Supplement: Supplementary file 1 — Additional file 1: Supplement Figure 1. CD34expression within cell clusters in human wound tissue. Expression of CD34 in fibroblast cell clusters and proportion of cells originating from diabetic foot ulcer (DFU) healers compared to non-healers for each cluster. Left panel: CD34 expression in CD34- and CD34+ fibroblast cell clusters split by wound healing response. Right panel: Expression of CD34 in all identified fibroblast subpopulations showing high CD34 expression in three clusters (Fb_2, Fb_6, Fb_7). Data are shown as violin plots. Supplement Figure 2. CD34+ and CD31+ cells repopulate human granulation tissue. Immunohistochemistry of a representative section of granulation tissue from a chronic lower limb wound in a 53-year-old male patient stained for CD34 (pink) and CD31 (brown). Left image: Cross-sectional view of the granulation tissue specimen. Scale bar indicates 1 mm. Right image: Detailed view of the same specimen showing the distribution of CD34+ cells (black arrows). Scale bars indicate 100 µm. Supplement Figure 3. Flow cytometry gating strategy. Flow cytometry gating strategy that discriminates single cell populations to define CD45-/CD31-/CD90+/CD34+ adipocyte precursor cells. Supplement Figure 4. Flow cytometric analysis. Flow cytometric analysis of single cell suspensions from human granulation tissue samples (n = 6). Mean frequencies of cell types are shown as a percentage of total viable granulation tissue cells. Supplement Figure 5. Effect of MQ-CM on ASC size and viability. Data from ASCs cultured in differentially activated MQ-CM for 72 h are shown as fold change compared to monocyte-CM treatment (Mo) (n = 5). Cell volume and diameter were analyzed using CASY TT cell counter. Cell viability corresponds to the number of cells negative for Annexin V and 7AAD staining as assessed by flow cytometry. Data are shown as mean ± SEM. Asterisks indicate p-values of <0.01 (**). Supplement Figure 6. Comparison of IL1B- and TGFB1-mediated effects on ASC p [file 41232_2023_302_MOESM1_ESM.pdf]

**Supporting Information for:**

# Preadipocytes in human granulation tissue: Role in wound healing and response to macrophage polarization

Tina Rauchenwald[1], Florian Handle[2], Catherine E. Connolly[1], Antonia Degen[1], Christof Seifarth[3], Martin Hermann[4], Christoph Tripp[5], Doris Wilflingseder[6], Susanne Lobenwein[1], Dragana Savic[7], Leo Pölzl[8], Evi M. Morandi[1], Dolores Wolfram[1], Ira-Ida Skvortsova[7], Patrizia Stoitzner[5], Johannes Haybaeck[2,9], Marko Konschake[3], Gerhard Pierer[1], Christian Ploner[1,*]

[1] Department of Plastic, Reconstructive and Aesthetic Surgery, Medical University of Innsbruck, Innsbruck, Austria
[2] Institute of of Pathology, Medical University Innsbruck, Innsbruck, Austria
[3] Department of Anatomy, Medical University of Innsbruck, Innsbruck, Austria
[4] Department of Anesthesiology and Critical Care Medicine, Medical University of Innsbruck, Innsbruck, Austria
[5] Department of Dermatology, Venerology and Allergology, Medical University of Innsbruck, Innsbruck, Austria
[6] Institute of Hygiene and Medical Microbiology, Medical University of Innsbruck, Innsbruck, Austria
[7] Department of Therapeutic Radiology and Oncology, Medical University of Innsbruck; EXTRO-Lab, Tyrolean Cancer Research Institute, Innsbruck, Austria
[8] Department of Cardiac Surgery, Medical University of Innsbruck, Innsbruck, Austria
[9] Diagnostic and Research Center for Molecular BioMedicine, Institute of Pathology, Medical University Graz, Graz, Austria

***Corresponding Author:** Christian Ploner, PhD
Department of Plastic, Reconstructive and Aesthetic Surgery
Medical University of Innsbruck, Austria
Email: christian.ploner@i-med.ac.at

Table of contents:

1) Supplement Figure 1
2) Supplement Figure 2
3) Supplement Figure 3
4) Supplement Figure 4
5) Supplement Figure 5
6) Supplement Figure 6
7) Supplement Figure 7
8) Supplement Table 1
9) Supplement Table 2

***Supplement Figure 1:*** **CD34 expression within cell clusters in human wound tissue.** Expression of CD34 in fibroblast cell clusters and proportion of cells originating from diabetic foot ulcer (DFU) healers compared to non-healers for each cluster. Left panel: CD34 expression in CD34- and CD34+ fibroblast cell clusters split by wound healing response. Right panel: Expression of CD34 in all identified fibroblast subpopulations showing high CD34 expression in three clusters (Fb_2, Fb_6, Fb_7). Data are shown as violin plots.

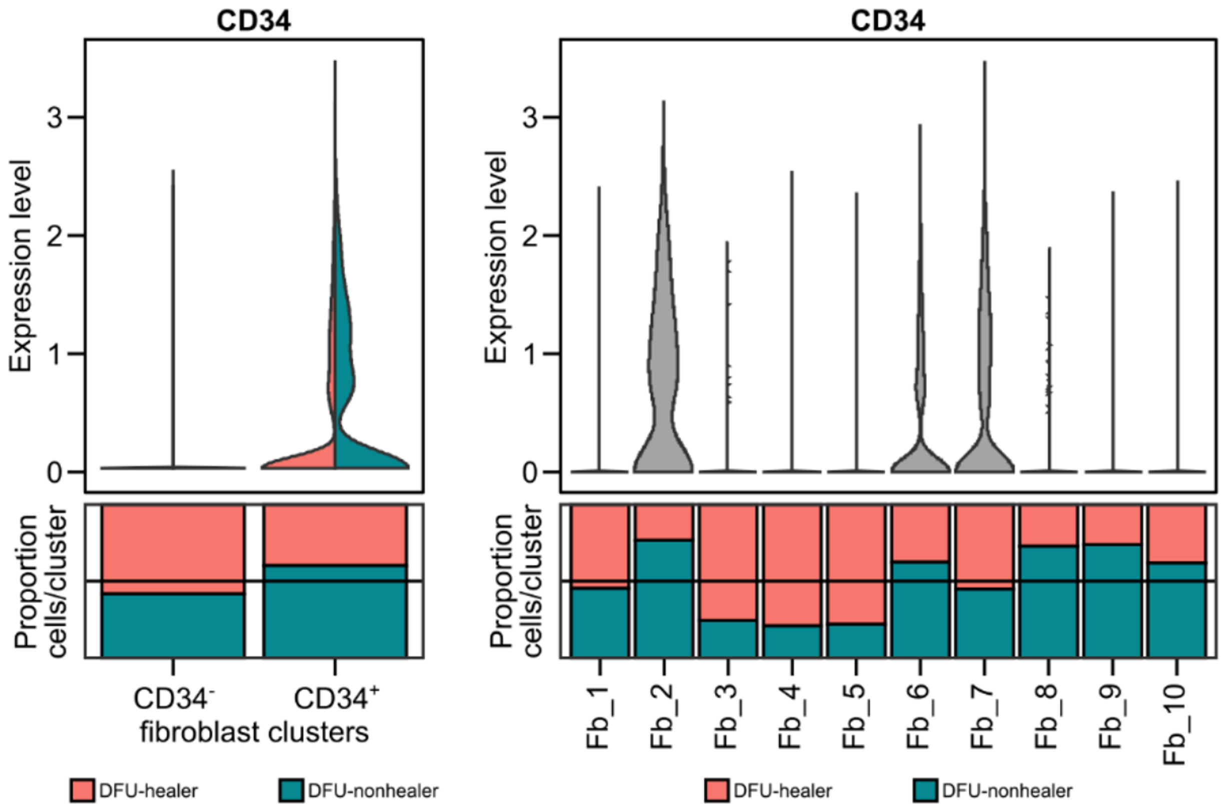

***Supplement Figure 2:*** **CD34+ and CD31+ cells repopulate human granulation tissue.** Immunohistochemistry of a representative section of granulation tissue from a chronic lower limb wound in a 53-year-old male patient stained for CD34 (pink) and CD31 (brown). Left image: Cross-sectional view of the granulation tissue specimen. Scale bar indicates 1 mm. Right image: Detailed view of the same specimen showing the distribution of CD34+ cells (black arrows). Scale bars indicate 100 µm.

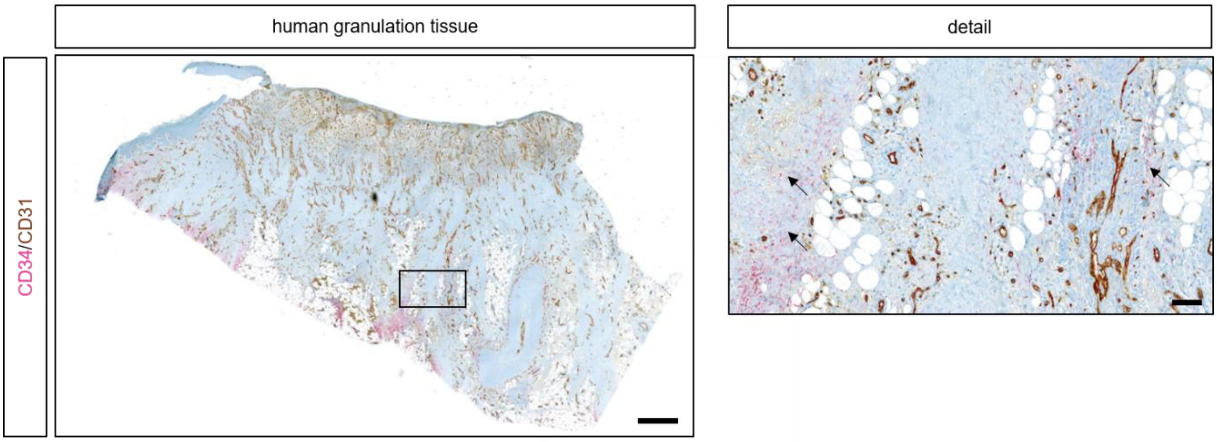

***Supplement Figure 3:*** **Flow cytometry gating strategy.** Flow cytometry gating strategy that discriminates single cell populations to define CD45-/CD31-/CD90+/CD34+ adipocyte precursor cells.

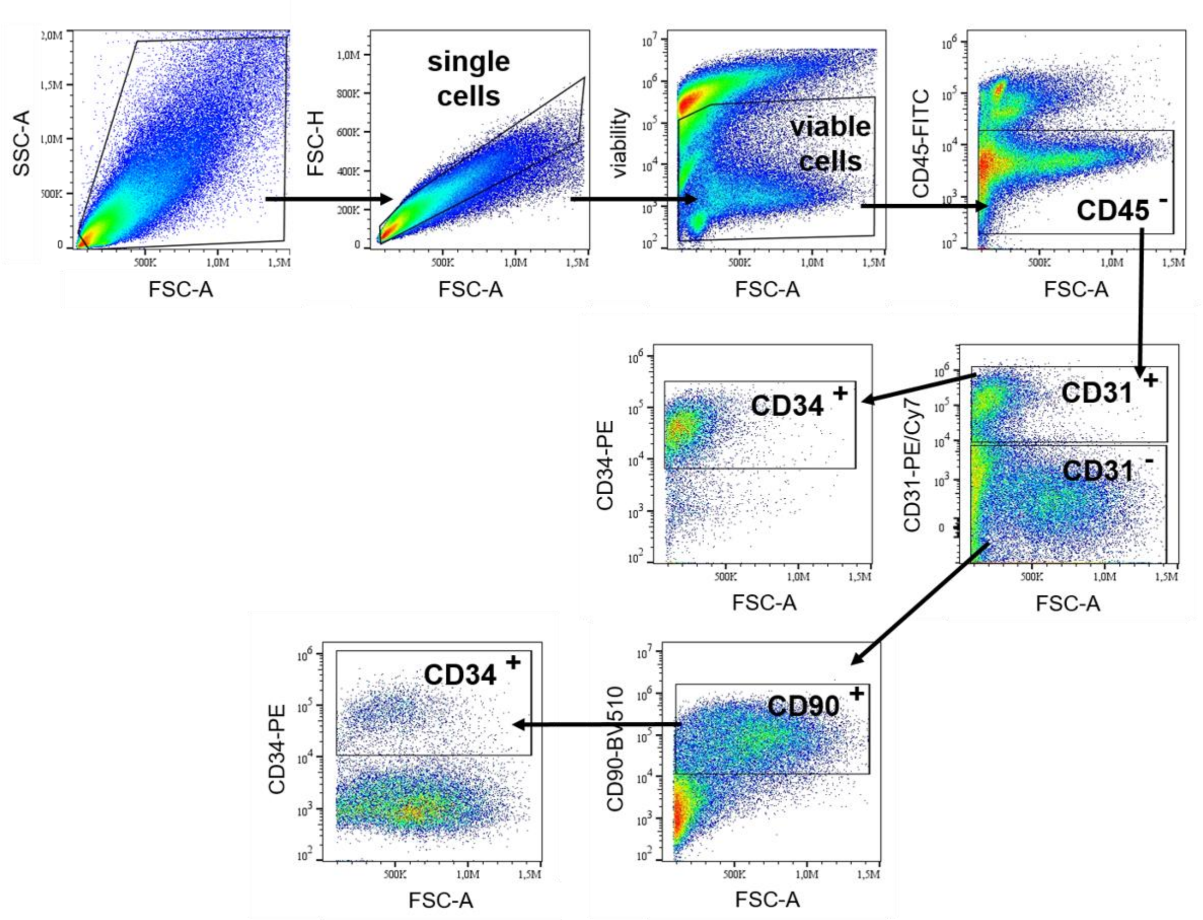

***Supplement Figure 4:*** **Flow cytometry analysis.** Flow cytometric analysis of single cell suspensions from human granulation tissue samples (n=6). Mean frequencies of cell types are shown as a percentage of total viable granulation tissue cells.

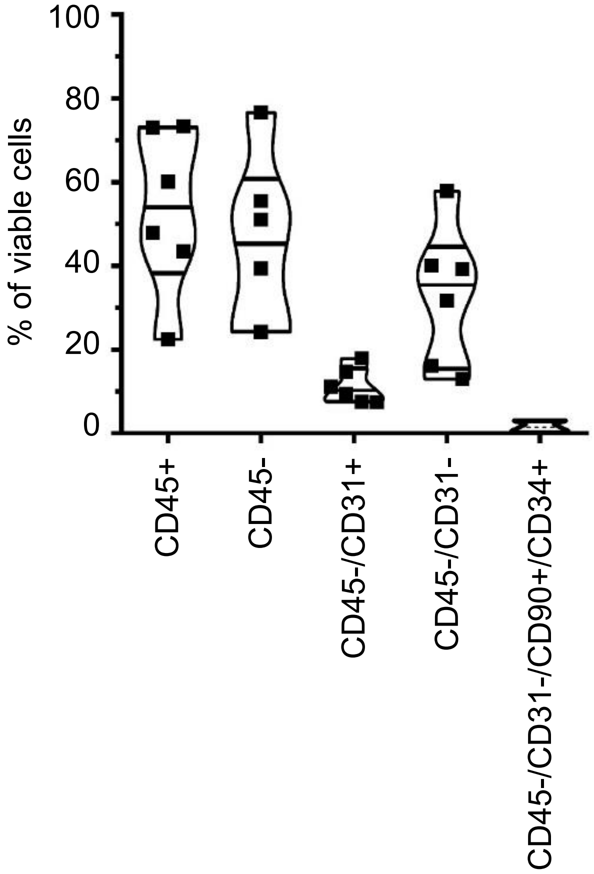

***Supplement Figure 5:*** **Effect of MQ-CM on ASC size and viability.** Data from ASCs cultured in differentially activated MQ-CM for 72 h are shown as fold change compared to monocyte-CM treatment (Mo) (n=5). Cell volume and diameter were analyzed using CASY TT cell counter. Cell viability corresponds to the number of cells negative for AnnexinV and 7AAD staining as assessed by flow cytometry. Data are shown as mean ± SEM. Asterisks indicate p-values of <0.01 (**).

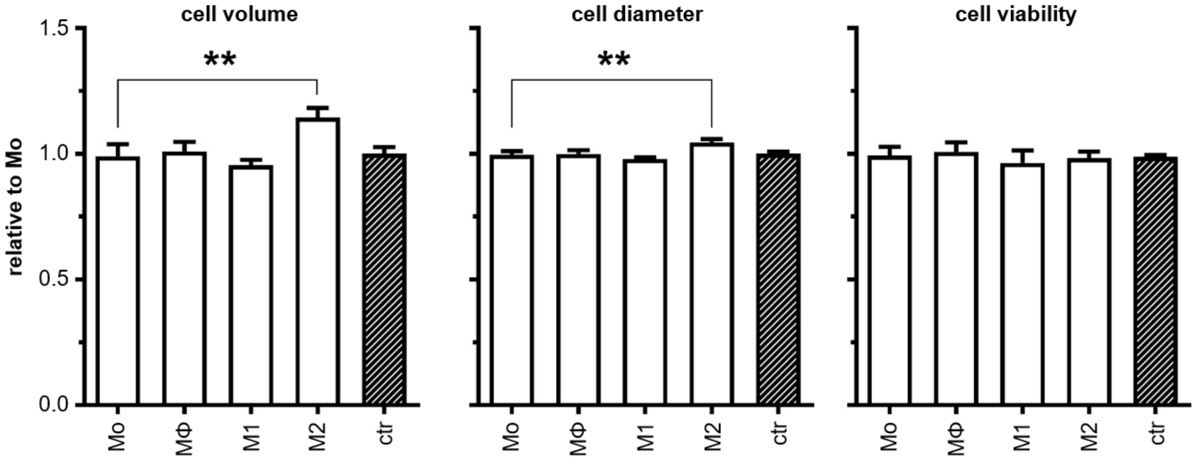

***Supplement Figure 6:*** **Comparison of IL1B- and TGFB1-mediated effects on ASC physiology.** (A) Representative confocal microscopy images of ASCs cultured in the presence of recombinant human IL1B (10 ng/ml) or TGFB1 (10 ng/ml) for 72 h and analyzed for actin cytoskeleton remodeling. Scale bars indicate 20 µm. (B) Quantification of mitochondrial membrane potential (MMP) (n=4). Data are shown as mean ± SEM. Asterisks indicate p-values of p<0.01 (**). (C-E) Representative immunoblots of cell lysates of the respective cells analyzed for cell adhesion proteins (C), intracellular kinases (D), or glycolysis regulating enzymes (E).

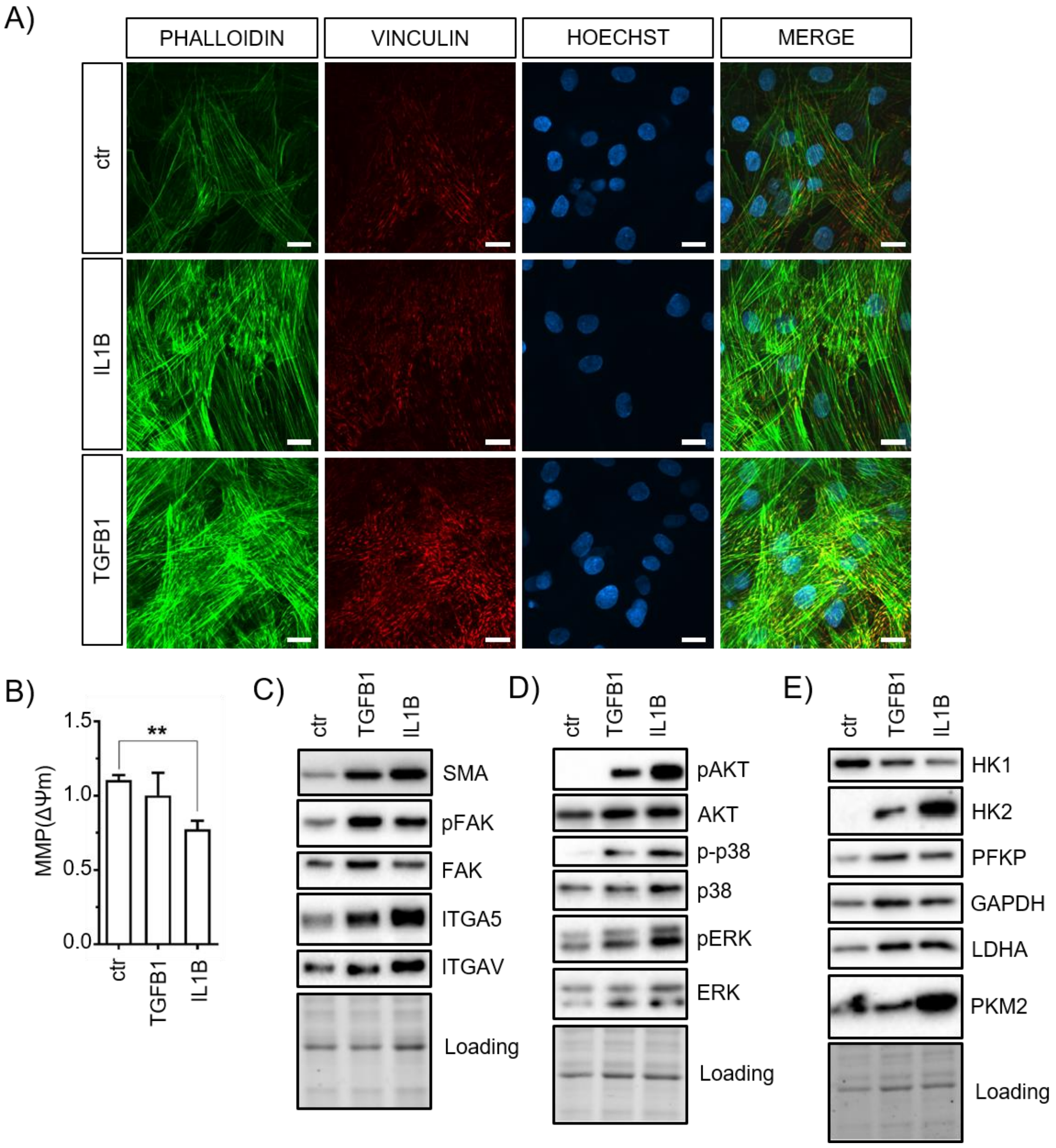

***Supplement Figure 7:*** **Efficacy of kinase inhibition.** (A) Representative immunoblots of ASCs exposed to different MQ-CM for 1 h. Kinase inhibition of M1-CM treatment with kinase inhibitors Ly294001 (PI3K/AKT), U0126 (ERK1/2), and Losmapimod (p38-MAPK) demonstrated efficacy. (B) Representative microscopic images of phalloidin staining (green) for actin stress fibers in ASCs cultured in M1-CM supplemented with LY294001 (10 µM), U0126 (10 µM) or Losmapimod (10 µM) for 72 h.

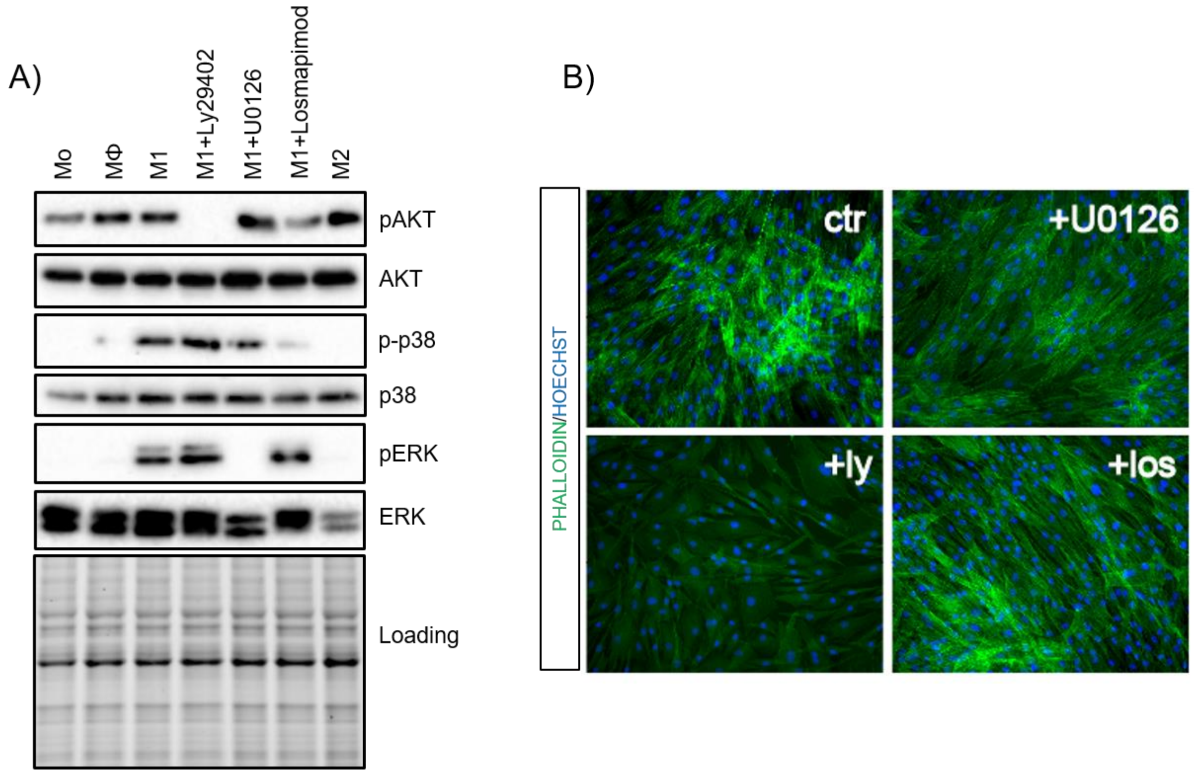

***Supplement Table 1.***

Chronic wound patient characteristics.

| Patient No. | Age | Sex | Wound type | Localization |
|---|---|---|---|---|
| 1 | 42 | male | pressure ulcer | ischial tuberosity |
| 2 | 18 | male | pressure ulcer | greater trochanter |
| 3 | 68 | male | pressure ulcer | ischial tuberosity |
| 4 | 18 | female | pressure ulcer | ischial tuberosity |
| 5 | 70 | female | pressure ulcer | ischial tuberosity |
| 6 | 44 | male | pressure ulcer | ischial tuberosity |

***Supplement Table 2.***

*List of Primers used for quantitative RT-PCR.*

| Gene | Sense primer (5'-3') | Antisense primer (5'-3') |
|---|---|---|
| **human CCL5** | GAGGCTTCCCCTCACTATCC | CTCAAGTGATCCACCCACCT |
| **human COL1A1** | ATGCCTGGTGAACGTGGT | AGGAGAGCCATCAGCACCT |
| **human COL3A1** | CTGGACCCCAGGGTCTTC | CATCTGATCCAGGGTTTCCA |
| **human COL4A1** | CAGCCAGACCATTCAGATCC | TGGCGCACTTCTAAACTCCT |
| **human COL6A1** | GACCTCGGACCTGTTGGGTAC | TACCCCATCTCCCCCTTCAC |
| **human COL7A1** | CGGAACTGACCATCCAGAAT | AATAGGGTGCTCACGGTCAC |
| **human FGF2** | GGCTTCTTCCTGCGCATCCAC | GGTAACGGTTAGCACACACTCC |
| **human FN1** | TGGACCAAGTTGATGACACC | CACCAGGTTGCAAGTCACTG |
| **human HGF** | AAGGTGACTCTGAATGAGTC | GGCACATCCACGACCAGGAACAATG |
| **human IL1B** | ACAGATGAAGTGCTCCTTCCA | GTCGGAGATTCGTAGCTGGAT |
| **human IL6** | AGACAGCCACTCACCTCTTCAG | TTCTGCCAGTGCCTCTTTGCTG |
| **human IL8** | ATGACTTCCAAGCTGGCCGTGGCT | TCTCAGCCCTCTTCAAAAACTTCTC |
| **human IP10** | CCTTATCTTTCTGACTCTAAGTGGC | ACGTGGACAAAATTGGCTTG |
| **human KGF** | TCCTGCCAACTTTGCTCTACA | CAGGGCTGGAACAGTTCACAT |
| **human LAMA5** | GGTGTGTCTCTGCGTGACAA | CCCCGACGTAGAAGACGAA |
| **human MCP1** | GTCTTGAAGATCACAGCTTCTTTG | AGCCAGATGCAATCAATGCC |
| **human MMP1** | GGGAGATCATCGGGACAACTC | GGGCCTGGTTGAAAAGCAT |
| **human MMP9** | GGTGATTGACGACGCCTTTGC | CGCGACACCAAACTGGATGAC |
| **human PDGFA** | CCTGCCCATTCGGAGGAAGAG | TTGGCCACCTTGACGCTGCG |
| **human TGFA** | AGGTCCGAAAACACTGTGAGT | AGCAAGCGGTTCTTCCCTTC |
| **human VEGF** | GAGGAGGGCAGAATCATCACGA | GTTTAACTCAAGCTGCCTCGCC |
| **human 18S rRNA** | GCAATTATTCCCCATGAACG | GGCCTCACTAAACCATCCAA |
