# Supplementary material for: Preadipocytes in human granulation tissue: role in wound healing and response to macrophage polarization
Source: Inflamm Regen. 2023 Oct 31;43:53. doi: 10.1186/s41232-023-00302-5 (PMC10617061; doi:10.1186/s41232-023-00302-5)

**Supporting Information: Original full length images of immunoblots**

# Preadipocytes in human granulation tissue: Role in wound healing and response to macrophage polarization

Tina Rauchenwald[1], Florian Handle[2], Catherine E. Connolly[1], Antonia Degen[1], Christof Seifarth[3], Martin Hermann[4], Christoph Tripp[5], Doris Wilflingseder[6], Susanne Lobenwein[1], Dragana Savic[7], Leo Pölzl[8], Evi M. Morandi[1], Dolores Wolfram[1], Ira-Ida Skvortsova[7], Patrizia Stoitzner[5], Johannes Haybaeck[2,9], Marko Konschake[3], Gerhard Pierer[1], Christian Ploner[1,*]

[1] Department of Plastic, Reconstructive and Aesthetic Surgery, Medical University of Innsbruck, Innsbruck, Austria

[2] Institute of Pathology, Neuropathology and Molecular Pathology, Medical University Innsbruck, Innsbruck, Austria

[3] Department of Anatomy, Medical University of Innsbruck, Innbruck, Austria

[4] Department of Anesthesiology and Critical Care Medicine, Medical University of Innsbruck, Innsbruck, Austria

[5] Department of Dermatology, Venerology and Allergology, Medical University of Innsbruck, Innsbruck, Austria

[6] Institute of Hygiene and Medical Microbiology, Medical University of Innsbruck, Innsbruck, Austria

[7] Department of Therapeutic Radiology and Oncology, Medical University of Innsbruck; EXTRO-Lab, Tyrolean Cancer Research Institute, Innsbruck, Austria

[8] Department of Cardiac Surgery, Medical University of Innsbruck, Innsbruck, Austria

[9] Diagnostic and Research Center for Molecular BioMedicine, Institute of Pathology, Medical University Graz, Graz, Austria

***Corresponding Author:** Christian Ploner
Department of Plastic, Reconstructive and Aesthetic Surgery
Medical University of Innsbruck, Austria
Email: christian.ploner@i-med.ac.at

**Description**

In the supplemental information the original uncropped merged images are shown as full length blots. Signals were acquired using a ChemidocMP gel analyzer. Shown are uncropped merged images of the chemiluminescence signal (antibody signal) and white light acquisition (membrane image, sizemarker). Unprocessed images were merged using Image Lab software (Version 5.2.1).

**Full-length unedited images for Figure 2F**

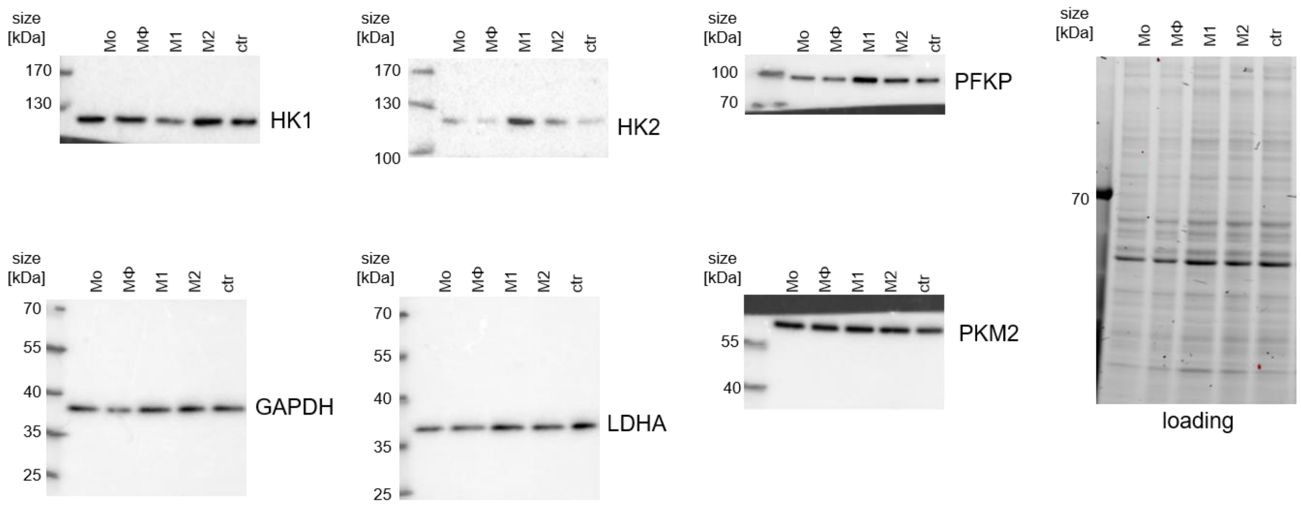

# Full-length unedited images for Figure 2G

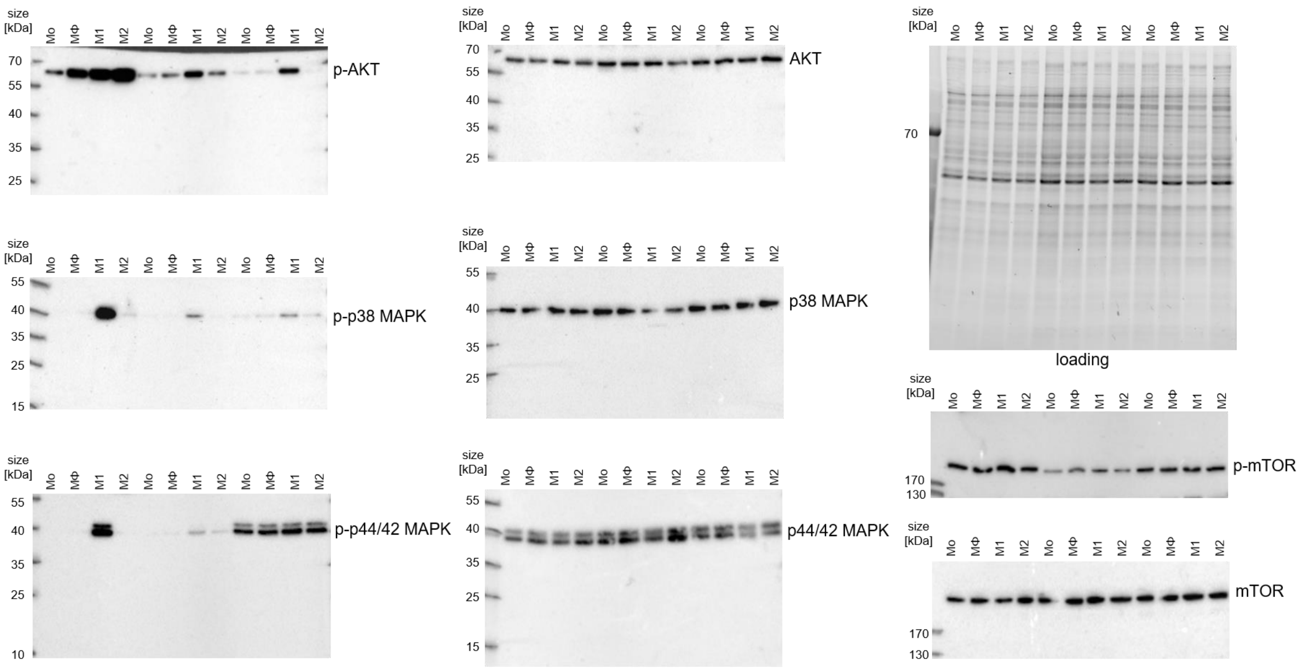

**Full-length unedited images for Figure 3B**

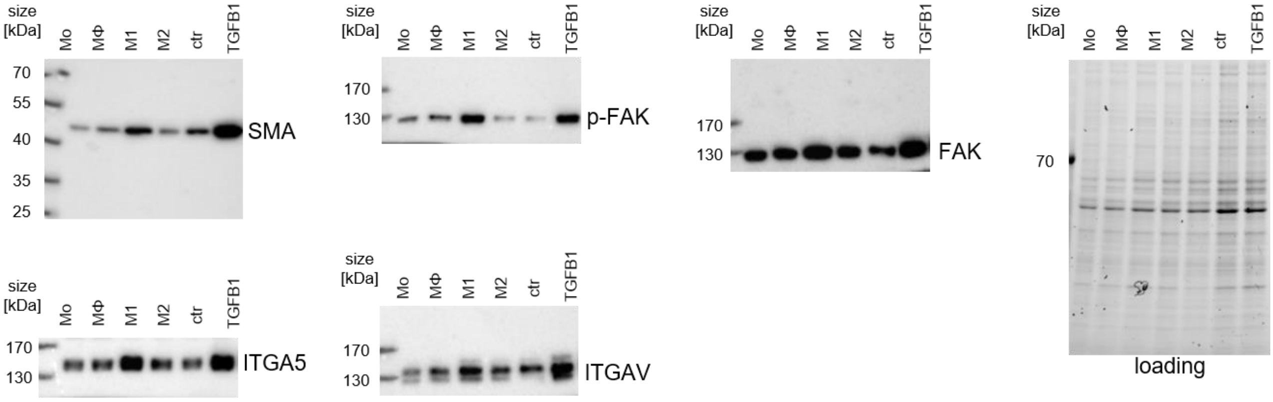

## Full-length unedited images for Figure 4B

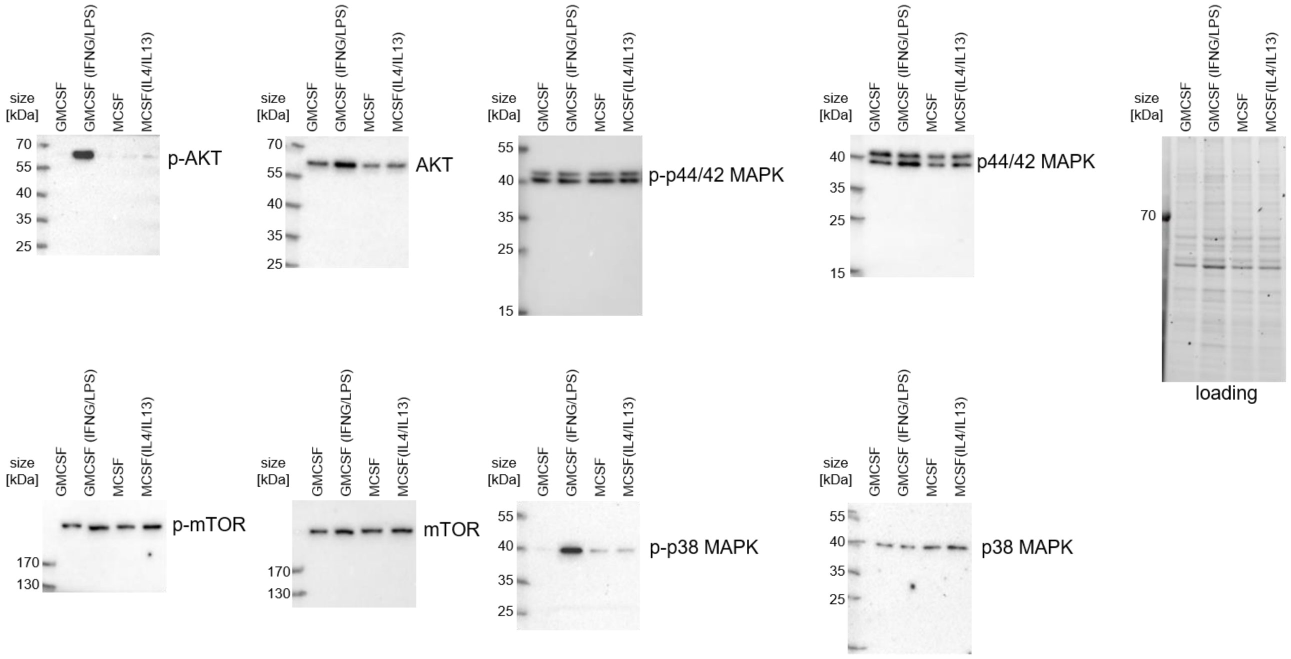

# Full-length unedited images for Figure 4C

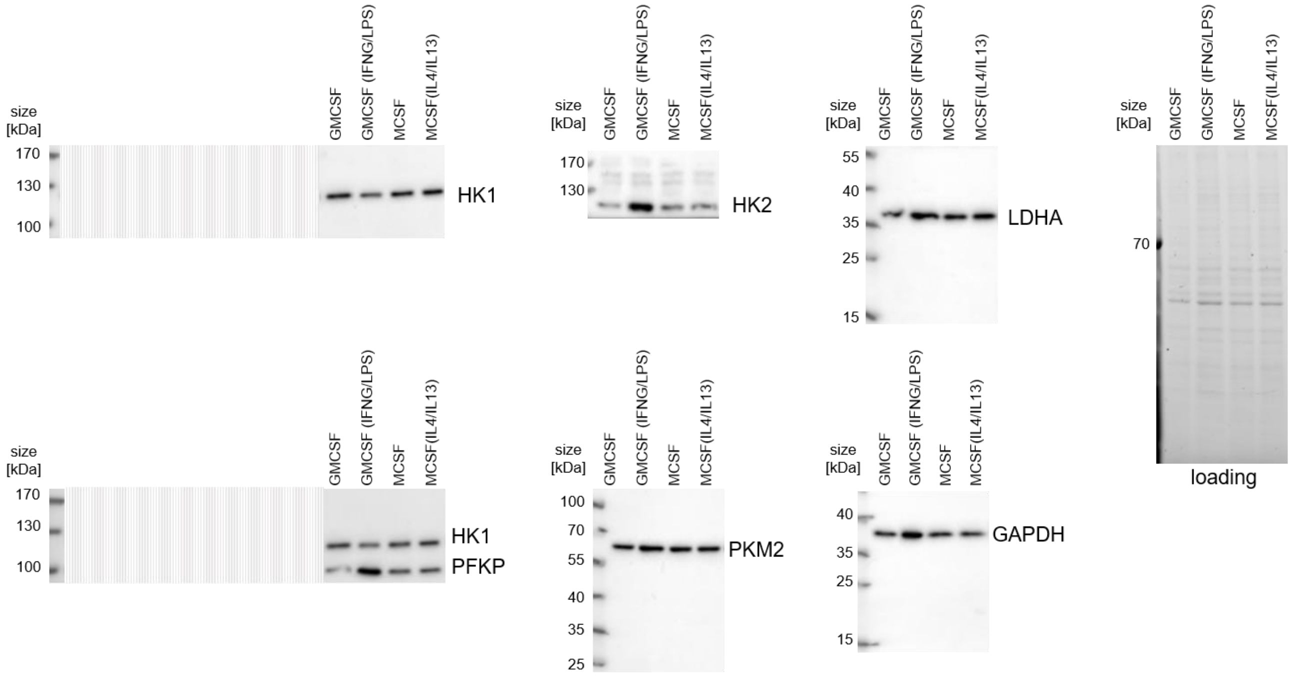

## Full-length unedited images for Figure 4D

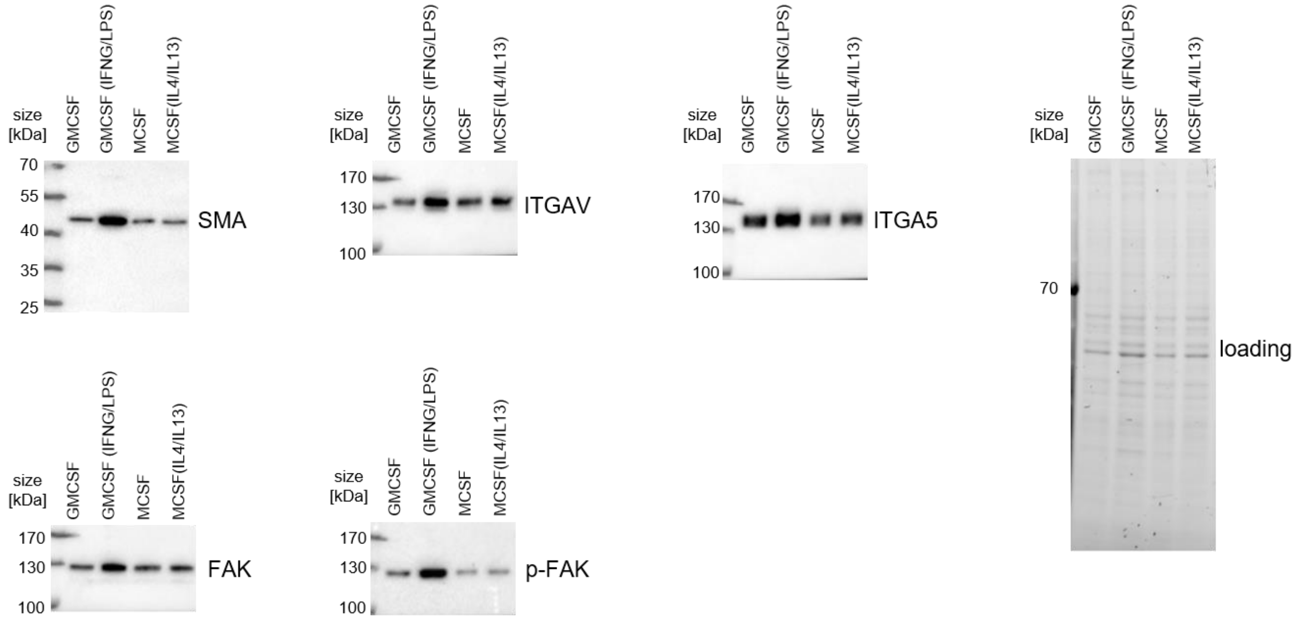

**Full-length unedited images for Figure 6B**

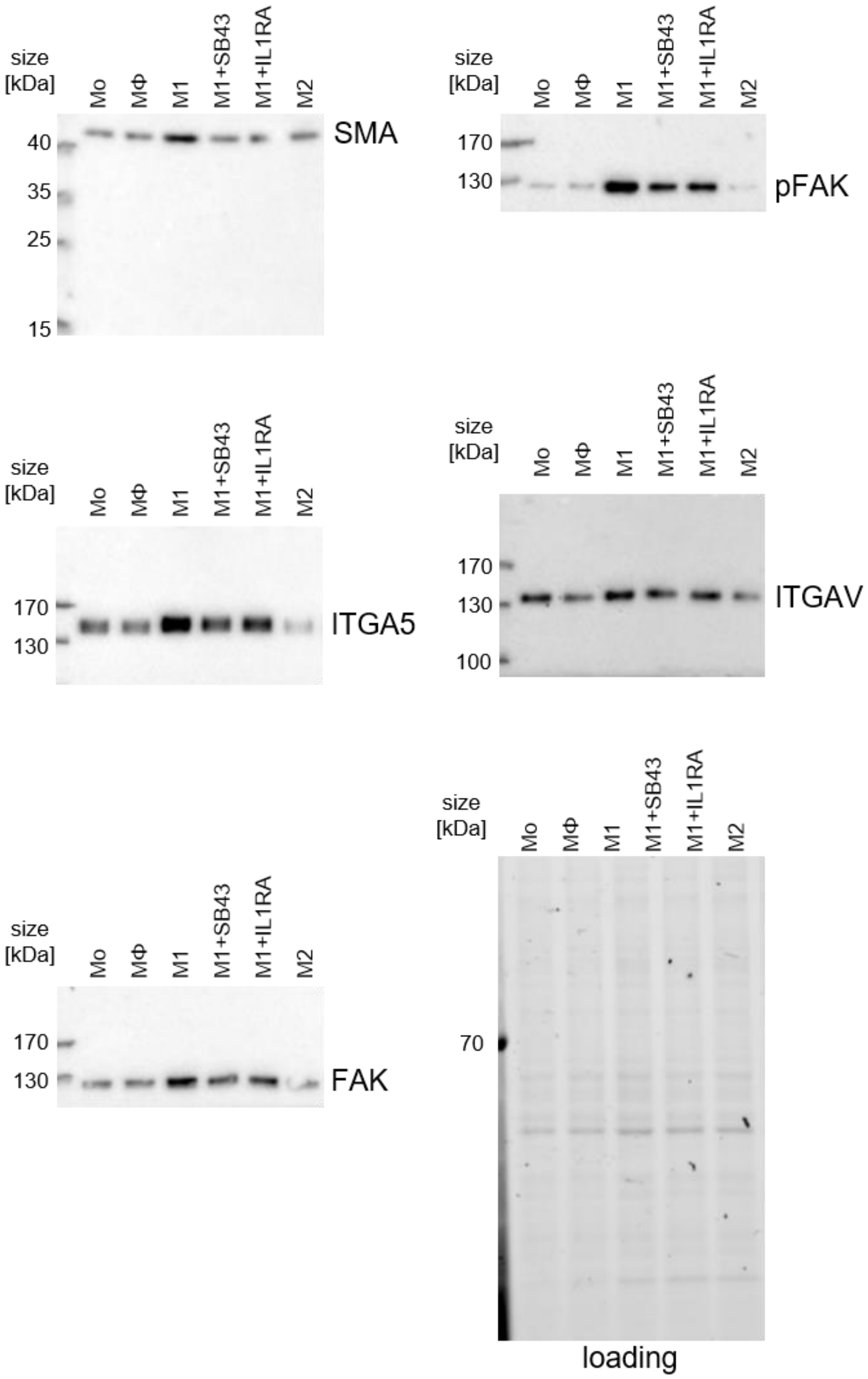

## Full-length unedited images for Figure 6C

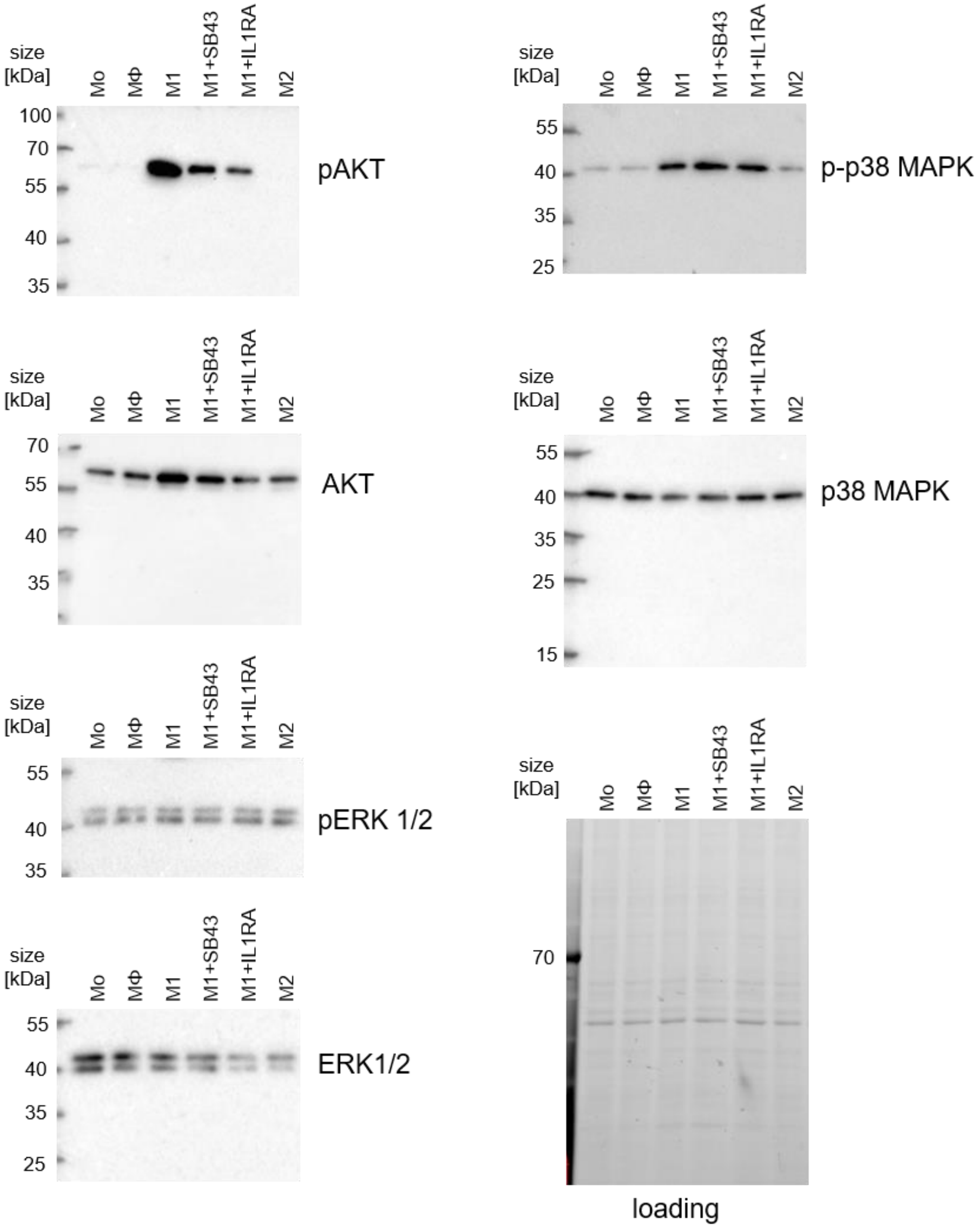

**Full-length unedited images for Figure 6F**

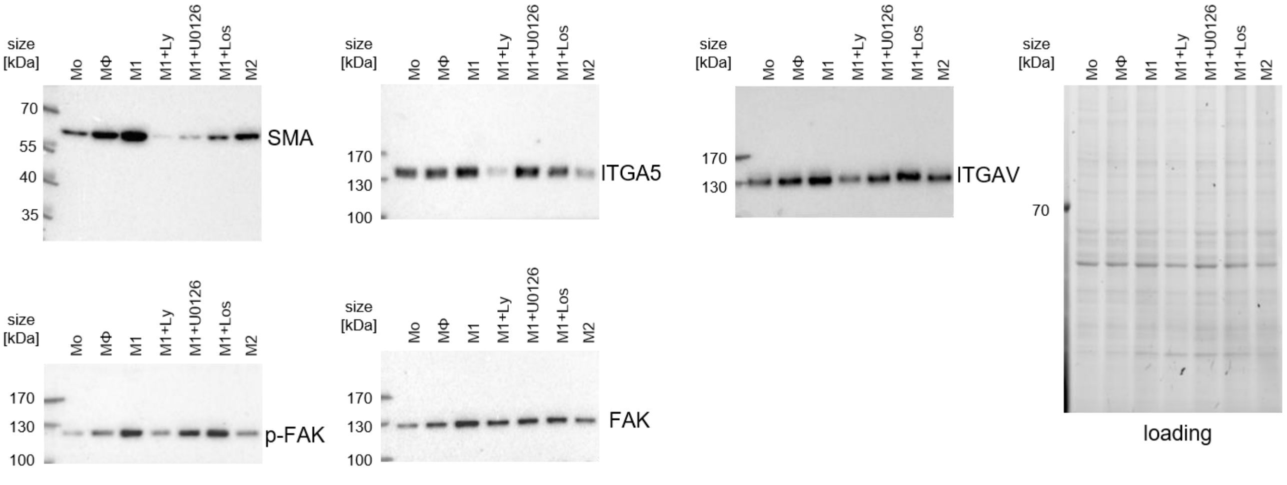

Supplement: Supplementary file 2 — Additional file 2: Supplement Table 1. Chronic wound patient characteristics. Supplement Table 2. Quantitative RT-PCR primers. All Primers used in this study are listed in the table below. [file 41232_2023_302_MOESM2_ESM.pdf]
